# Enrichment of electrotrophic microorganisms from contrasting shallow-sea hydrothermal environments in bioelectrochemical reactors

**DOI:** 10.3389/fmicb.2025.1539608

**Published:** 2025-02-03

**Authors:** Antoine Carissimo, Victoria Comes, Alenica Heussner, Anne-Hélène Prime, Roy E. Price, Gaël Erauso, Pierre-Pol Liebgott, Sven Kerzenmacher, Guillaume Pillot

**Affiliations:** ^1^Center for Environmental Research and Sustainable Technology (UFT), University of Bremen, Bremen, Germany; ^2^Aix Marseille Univ, Université de Toulon, CNRS, IRD, MIO, Marseille, France; ^3^School of Marine and Atmospheric Sciences, Stony Brook University, Stony Brook, NY, United States

**Keywords:** hydrothermal vents, electrotrophs, extremophiles, thermophiles, alkaliphiles, bioelectrochemical reactors, microbial electrosynthesis

## Abstract

**Introduction:**

Hydrothermal vents are inhabited by electrotrophic microorganisms, which are capable of oxidizing extracellular compounds, such as metals, to power their metabolisms. However, their diversity is poorly known, especially in shallow-sea hydrothermal vents where it has not been extensively studied. Bioelectrochemical reactors can be used to investigate such electrotrophic diversity by providing an electrode as an electron donor.

**Methods:**

Here, a total of 60 different reactors were set up and inoculated with either a microbial community coming from the shallow, acidic (ca. pH 5.5) and hot (ca. 120°C) hydrothermal system of Panarea, Aeolian islands, Italy, or the shallow, alkaline (pH 11) and mild (40°C) hydrothermal system of Prony Bay, New Caledonia.

**Results:**

With the alkaline sample, no electrical current increase was seen in any of the 15 reactors operated for 6 days under Prony hydrothermal conditions (pH 10, 30–75°C). By contrast, a 6-fold increase on average was observed in reactors operated under the Panarea hydrothermal fluid conditions (pH 4.5–7, 75°C). A Multi-Factor Analysis revealed that the overall bioelectrochemical performances of these reactors set them apart from all the other Panarea and Prony conditions, not only due to their higher current production but also archaeal abundances (measured through qPCR). Most reactors produced organic acids (up to 2.9 mM in 6 days). Still, coulombic efficiencies indicated that this might have been due to the (electro) fermentation of traces of yeast extract in the medium rather than CO_2_ fixation. Finally, microbial communities were described by 16S metabarcoding and ordination methods, and potential electrotrophic taxa were identified. In Panarea reactors, higher growth was correlated with a few bacterial genera, mainly *Bacillus* and *Pseudoalteromonas*, including, for the former, at higher temperatures (55°C and 75°C). In reactors reproducing the Prony Bay hydrothermal conditions, known facultative methylotrophs, such as *Sphingomonas* and *Methylobacterium*, were dominant and appeared to consume formate (provided as carbon source) but no electrons from the cathode.

**Conclusion:**

These results provide new insights into the distribution and diversity of electrotrophs in shallow-sea hydrothermal vents and allow the identification of potential novel biocatalysts for Microbial Electrosynthesis whereby electricity and carbon dioxide are converted into value-added products.

## 1 Introduction

In hydrothermal vents, microorganisms can thrive by exploiting the geochemical energy provided by the reduced hydrothermal fluid as it is discharged in the oxidizing seawater environment. Different microbial physiologies are supported by the conditions in and around the vents, from hyperthermophiles (optimal growth temperature > 80°C) and acidophiles or alkaliphiles thriving in the chimneys, to psychrophiles and neutrophiles in the surrounding seawater (Kelley et al., 2005; Dick, 2019). Various microbial metabolisms have been characterized, including chemolithoautotrophs, which form the trophic basis of these chemosynthetic ecosystems (Sievert and Vetriani, 2012; Dick, 2019).

The composition and properties of a hydrothermal fluid differ based on the flow path and type of rock through which the water circulates and interacts with minerals, as well as the heat source (Kelley et al., 2002; Hannington et al., 2005; Dick, 2019). At least two general types of vents can be distinguished: vents formed by the proximity of a magmatic chamber and vents formed by an exothermic water-rock reaction called serpentinization. In the former, the fluid is usually acidic (down to pH 2) and very hot (up to about 400°C). During serpentinization, however, Fe(II)-bearing minerals (e.g., olivine) in ultramafic rocks reduce water to form abundant H_2_, leading to an alkaline (pH up to 11), moderately warm (up to 90°C in Lost City) fluids (Kelley et al., 2005).

Hydrothermal ecosystems provide various electron donors and acceptors to power metabolisms, including metals from the fluids (Zeng et al., 2021). Microorganisms using extracellular, insoluble compounds such as metals for energy are termed electroactive microorganisms (Lovley and Holmes, 2021). Among them, exoelectrogens can reduce extracellular electron acceptors, while electrotrophs can oxidize extracellular electron donors. Hydrothermal vents are known to harbor a large diversity of electroactive microorganisms (Vargas et al., 1998; Lovley and Holmes, 2021; Zeng et al., 2021).

In addition to metals, microbial cells, and other chemical compounds, electrotrophs can use an electrode as an electron donor (cathode) in bioelectrochemical systems. When carbon dioxide (CO_2_) is used as the sole carbon source, this metabolism is termed electroautotrophy and is the basis for Microbial Electrosynthesis (ME) (Rabaey and Rozendal, 2010; Tremblay et al., 2017). The biotechnological potential of these microorganisms resides in their ability to catalyze electrochemical reactions of interest, such as reducing CO_2_ into value-added products (e.g., acetic acid and methane) (Rabaey and Rozendal, 2010). Different metabolic types from diverse environments can grow electrolithoautotrophically, including acetogens, methanogens, iron-oxidizers and-reducers, sulfate-reducers, sulfur-oxidizers, nitrate-reducers, and phototrophs (Gupta et al., 2020). However, most biocatalysts tested for ME so far are mesophilic acetogens and methanogens, with acetate and methane as the main products, respectively. Studying the diversity of electrotrophs could allow for the discovery of novel microbial metabolisms suitable for ME, possibly broadening the spectrum of products from CO_2_ reduction in ME. Increasing the temperature of ME would also have several advantages, including faster reaction rates and lower contamination risks, but only a few thermophiles have been tested so far (Dopson et al., 2016; Shrestha et al., 2018; Jourdin and Burdyny, 2021; Chaudhary et al., 2022).

Interestingly, natural electrical currents have been discovered in recent years across deep-sea hydrothermal chimneys, and it has been suggested that microorganisms colonizing such structures could directly feed on these electrical currents (Yamamoto et al., 2018). Using a cathode as a primary electron source in a bioelectrochemical reactor allows targeting of these electroautotrophic microorganisms for specific enrichment (Rowe et al., 2015). With this approach, hyperthermophilic communities were enriched in the laboratory (Pillot et al., 2021), while mesophilic communities were enriched *in situ* (Yamamoto et al., 2023). Recently, it was shown that pure cultures of hyperthermophilic bacteria and archaea, isolated from hydrothermal environments, can grow with a cathode in electroautotrophic conditions (Popall et al., 2022), suggesting that electroautotrophy is a common metabolism in hydrothermal vents. To confirm this, different types of hydrothermal systems should be investigated, including magmatism-based and serpentinite-hosted vents. Both types have been shown to occur as deep-sea hydrothermal vents (DHVs) and shallow-sea hydrothermal vents (SHVs), which are typically defined as occurring in less than 200 m water depth (Price and Giovannelli, 2017).

Compared with DHVs, the geochemistry and microbiology of SHVs are generally less studied (Price and Giovannelli, 2017). Whether natural electric currents occur in those environments is currently unknown. Nevertheless, numerous chemolithoautotrophs have been isolated from SHVs (Price and Giovannelli, 2017). As with DHVs, their physiology is adapted to the extreme conditions prevailing in the vicinity of the vents, including high temperatures and extreme pH values.

To investigate electrotrophic communities in SHVs and identify their preferred ecological niches, it is necessary to increase cultivation efforts to cover the extensive ranges of temperature, pH, and electron acceptors that support microbial growth in hydrothermal environments. In this study, two shallow-sea hydrothermal vent samples, harboring microbial communities, were used to inoculate 60 bioelectrochemical reactors: one sample from hot (ca. 120°C) and acidic (ca. pH 5.5) hydrothermal sediments off the coast of Panarea, Aeolian islands, Italy, and one sample of carbonate chimney from an alkaline vent (pH 11, 40°C) from Prony Bay, New Caledonia. For each sample, 3 temperatures, 2 pH values, and 5 electron acceptors were tested. The cathodic potential was fixed at-600 mV vs. SHE. Growth and electrochemical performances were monitored, and the enriched microbial communities were identified by DNA metabarcoding.

## 2 Materials and methods

### 2.1 Sample collection

In this study, two samples from two different hydrothermal systems were used: Panarea, Aeolian Islands, Italy, and Prony Hydrothermal Field, New Caledonia. The Aeolian Islands make up a volcanic arc, and the associated shallow-sea hydrothermal system is therefore influenced by magmatic activity resulting from plate tectonics. The discharged fluid at Panarea is anoxic, hot (up to 135°C), and acidic (as low as pH 1.9) (Italiano and Nuccio, 1991; Price et al., 2015). The “La Calcara” site is an area of heated sediments located at about 20 mbsl (meters below sea level) with diffuse hydrothermal gas (>98% CO_2_, 2% H_2_S; Italiano and Nuccio, 1991) and fluid venting, as described elsewhere (Price et al., 2015). The LC2 sample (stands for La Calcara number 2 sample) consists of coarse-grained sand collected by scuba diving at the La Calcara (latitude 38°38′40.7129″, longitude 15°04′32.6926″) in June 2017. The sediment-fluid mixture was collected using a small shovel and directly transferred to a 100 mL Pyrex flask (G45 Schott), which had been rinsed several times with the hydrothermal fluid percolating through the sediment during the dive, taking care to keep contamination with the surrounding seawater to a minimum. The completely filled flask was capped with a butyl septum (gas-tight), secured with a screw cap (G45) with a central opening, and brought to the surface boat, placed in a cooler, and brought to the Eccsel Panarea Natural Laboratory (https://www.eccsel.org/catalogue/124) on Panarea Island within 2–3 h. The sample was then kept at 4–5°C and then shipped in a refrigerated insulated box at 5°C to the institute lab, where it was kept at 5°C until use. A temperature of 120°C and a pH of 5.5 were measured *in situ* and within an hour after collection, respectively.

The Prony Bay Hydrothermal Field (New Caledonia) is a shallow coastal hydrothermal system with a different geochemical context. There, the anoxic hydrothermal fluid is generated by serpentinization (Launay and Fontes, 1985; Monnin et al., 2014, 2021). The fluid discharging at the bottom is hyperalkaline (up to pH 11.9), at mild temperature (max. 42°C), highly reduced (−650 mV), and enriched in H_2_, CH_4,_ and N_2_ in variable proportions (Monnin et al., 2014, 2021). At the ‘Bain des Japonais’ site, a shallow-sea spring uncovered at low tide, the venting gases contain between 4 and 41% of H_2_ and between 8 and 20% of CH_4_. The BJ sample is a piece of small carbonate chimney (~20 cm high). It was collected at about 0.3 mbsl, and the fluid had a temperature of 37–40°C, a pH of 11.1, and a redox potential (E_h_) of-237 mV. It was collected in November–December 2018 at 22°17.970’S, 166°51.708’E (Monnin et al., 2021). In the laboratory (Center IRD de Nouméa), a chimney subsample was crushed and transferred anaerobically to a hermetically sealed serum bottle with a thick butyl septum and screw lid, and stored at 4°C until use.

### 2.2 Culture conditions

As the hydrothermal fluid salt composition in LC2 is similar to seawater (Price et al., 2015), an artificial seawater medium (ASM), previously used to cultivate multiple hyperthermophilic hydrothermal vent isolates (Popall et al., 2022), was used in the experiments with the LC2 sample. The medium contained, per liter, 20 g of NaCl, 5 g of MgCl_2_.6H_2_0, 1 g of NaHCO_3_, 0.3 g of MgSO_4_.7H_2_0, 0.3 g of NH_4_Cl, 0.3 g of CaCl_2_.2H_2_O, 0.3 g of KCl, 0.3 g of KH_2_PO_4_, 0.2 g of yeast extract, 10 mL of Wolfe’s mineral elixir and 1 mL of vitamin solution (Wolin et al., 1963). Chemicals were purchased from Carl Roth, Germany. It was prepared in an anaerobic chamber (Coy Anaerobic Monitor CAM-12, Coy Lab Products, USA) using anaerobic deionized (DI) water. The medium was adjusted to pH 4.5 with 1 M HCl and then autoclaved at 120°C for 20 min. After autoclaving, the pH was adjusted to 4.5 when necessary (acid reactors) or increased to 6.8 with 1 M NaOH (neutral reactors). It was then stabilized with a 0.1 M phosphate buffer. MgCl_2_, CaCl_2_, MgSO_4_, and KH_2_PO_4_ salts were added after autoclaving from filter-sterilized concentrated solutions.

For experiments with the BJ sample, the basal medium, previously used to enrich indigenous alkaliphiles (Postec et al., 2021), contained per liter: 2.5 g of NaCl, 1.36 g of HCOONa (20 mM of formate), 0.16 g of K_2_HPO_4_, 0.1 g of NH_4_Cl, 0.1 g of MgCl_2_.6H_2_O, 0.1 g of yeast extract, 0.08 g of CaCl_2_.2H_2_O and 0.08 g of Na_2_S_2_O_3_, and 5 mL of Balch’s oligo-elements (Balch et al., 1979). Formate was provided as an alternative carbon source, as it was found in alkaline hydrothermal fluids of serpentinization-based environments (Lang et al., 2010) where inorganic carbon is poorly available due to high pH and precipitation in the relatively acidic surrounding freshwater or seawater in the presence of Ca and Mg ions. The medium was prepared under anaerobic conditions, with the pH adjusted to 7 or 10 with 1 M NaOH, depending on the experiment, before and after autoclaving.

### 2.3 Reactor setup and operation

H-cell type reactors were used. A total of 60 reactors were prepared: 30 inoculated with the LC2 sample and 30 with the BJ sample. For each sample, three temperatures (30°C, 55°C and 75°C), two pH (4.5 and 7 for LC2; 7 and 10 for BJ) and five electron acceptors (see below) were tested. The reactors were not replicated. A graphite plate (1.5 by 1.5 cm, MR40, Müller & Rössner, Troisdorf, Germany) was used as a working electrode (cathode), and a titanium-coated iridium tantalum oxide mesh (2 by 2 cm) (Platinode® Mixed metal oxide Anode 177, Umicore, Schwäbisch Gmünd, Germany) was used as counter electrode (anode). To increase the hydrophilicity of its surface, the working electrode was sonicated for 10 min in 70% isopropanol, followed by 10 min in DI water (Pillot et al., 2022a). Both electrodes were mounted on 0.25 mm (Sigma-Aldrich, Germany) or 1 mm titanium wires (chemPUR, Germany). The two chambers were separated by an anion exchange membrane (AMI, Membrane Internationals, USA). Both chambers were sealed with gas-tight septum plugs. A KCl-saturated calomel electrode (K10, Sensortechnik Meinsberg, Germany) was used as a reference electrode and was inserted in the cathode chamber.

H-cells were autoclaved dry without membranes and reference electrodes. These were sterilized with 70% isopropanol. The membranes, reference electrodes, media and inoculums were added to the H-cells in the anaerobic chamber. A piece of carbonate chimney (BJ) or a spoon of sulfidic sediments (LC2) was added to the BJ and LC2 reactors, respectively. Two hundred and fifty mL of sterile medium was added to cathodic and anodic chambers each. Depending on the experiment, the cathodic chamber was supplied with 20 mM of KNO_3_, NaSO_4_, Na_2_S_2_O_3_, or 3.2 mM of Fe^3+^ as ferrihydrite (an iron oxyhydroxide). These are common electron acceptors for microbial anaerobic respiration in marine environments. Ferrihydrite was prepared by adding 10 M NaOH to a solution of FeCl_3._ Abiotic control reactors were prepared which contained the LC2 or BJ media but were not inoculated. These reactors were supplemented with a mixture of electron acceptors (KNO_3_, NaSO_4_, Na_2_S_2_O_3_, and ferrihydrite) to assess if the abiotic current baseline varied over time. As no variation was observed over time, the current before inoculation in each condition was used as baseline. Experiments ran for 6 days in an Incudrive H incubator (Schuett-Biotec, Germany) at 30°C or 55°C or HerathermTM incubator (Thermo Scientific, Germany) at 75°C. These temperatures were chosen to target mesophiles, thermophiles, and hyperthermophiles, respectively. Although 75°C is lower than the *in situ* LC2 temperature (120°C), it is sufficient for most hyperthermophiles to grow (Stetter, 1999) and is safer for the reference electrode, electrical cables, and magnetic stirrer. In the experiments with the LC2 sample, the medium was continuously gassed at 100 mL/min with N_2_:CO_2_ gas (80:20) for anaerobic conditions and with N_2_:CO_2_:O_2_ gas (77.5:20:2.5) for microaerophilic conditions (in which case dioxygen is provided as the sole electron acceptor). In the experiments with the BJ sample, the carbon source was formate (20 mM), and the medium was continuously gassed at 100 mL/min only with 100% N_2_ for anaerobic conditions and with N_2_:O_2_ gas (97.5:2.5) for microaerophilic conditions. The electrodes were set to a fixed potential of −0.6 V vs. SHE using a potentiostat (IPS Elektroniklabor, PGU-MOD-500 mA, Germany) and the EcmWin software (IPS Elektroniklabor, Germany) and the current consumption was monitored across time (chronoamperometry). This potential was chosen because it showed the most biofilm formation in previous experiments with a thermophilic bacterial strain (Pillot et al., 2021, 2022b). Reference electrodes were checked before and after experiment to evaluate potential shift. A maximum of 20 mV shift was measured in the worst case. Samples were taken daily with a syringe and stored at −20°C. pH of the sample were measured daily and the pH was adjusted in the reactor if deviating by more than 1 pH unit.

### 2.4 Fluorescence microscopy

At the end of the experiment, the cathodes were stored in a solution of glutaraldehyde 3% in PBS 0.1 M at 4°C. Biofilms on the cathodes were observed using a fluorescence microscope (Axioscope 7, Carl Zeiss, Germany) and ZEN pro 3.0 software (Carl Zeiss, Germany). The graphite plates were first rinsed with deionized water and then stained with 2 μg/mL of 4′,6-diamidine-2-phenylindole (DAPI), 10 μg/mL acridine orange and 2 μg/mL Nile red (Carl Roth, Germany) in DI water for at least 15 min. Afterwards, the graphite plates were rinsed again with deionized water. Z-stacks were made at wavelengths of 385 nm (DAPI), 470 nm (acridine orange), and 565 nm (Nile red) at 250× magnification. Finally, images were deconvoluted and processed in ZEN 3.0.

### 2.5 Quantification of organic acids and chemical oxygen demand

Liquid samples taken during the experiment were centrifuged to remove particles and then analyzed by high-performance liquid chromatography (HPLC). An HPLC system equipped with 2,414 refractive and 2,489 UV index detectors (Waters, Germany), an Aminex HPX-87H column (Bio-Rad, USA) (35°C, 8 mM H_2_SO_4_ eluent at 0.6 mL/min), and controlled by the Empower® 3 software (Waters, Germany) was used.

The Chemical Oxygen Demand (COD) was measured in medium containing 0.1 or 0.2 g of yeast extract (Carl Roth, Germany) to establish an initial COD value for all reactors. It was then measured on the final sample of each reactor, using kits LCK 1914 for LC2 samples and LCK 514 for BJ samples (Hach, Germany), following manufacturer instructions for each specific salinity. All COD values can be found in Supplementary Figure S6. When the final COD in a given reactor was inferior to the initial COD, the difference was assumed to result from a microbial consumption of the yeast extract (expressed in coulombs). When the final COD was greater than the initial COD, it was hypothesized to be the result of electroautotrophic metabolisms (microbial CO_2_ fixation into biomass using electrons from the cathode).

### 2.6 Quantification of electron acceptors

Nitrate, nitrite, sulfate, and thiosulfate were quantified by ion chromatography. Samples were prepared by 100-fold dilution of the HPLC samples with DI water. Measurements were performed on an IC system (Metrohm, Germany) with a Metrosep A Supp-5150/4.0 separation column and Metrosep A Supp 4/5 Guard/4.0 precolumn (35°C, carbonate eluent containing 6.4 mM Na_2_CO_3_, 2 mM NaHCO_3_, 17 vol% acetone at 0.6 mL/min) using IC-Metrodata for Windows software (Metrohm, Germany).

Iron reduction was quantified by measuring the concentration of Fe(II) with the spectrophotometric ferrozine assay (Viollier et al., 2000) after sample dissolution in 2 M HCl.

### 2.7 Quantitative polymerase chain reaction (qPCR)

Procedures for qPCR were performed as described before (Pillot et al., 2022b). For qPCR, following fluorescence microscopy, the cathodes were treated with 0.1 M Tris–HCl in 0.1 M NH_4_Cl for 30 min to remove glutaraldehyde. They were then rinsed with DI water. Before performing qPCR, the WEs were sonicated for 10 min in 10 mL DI water to release the biofilm into the DI water. qPCR was then directly performed in triplicate in a 20 μL PCR mix on 2 μL of sample from the suspended biofilm, using archaeal (913F: AGG AAT TGG CGG GGG AGC A and 1100R: BGG GTC TCG CTC GTT RCC) and bacterial (GML5F: GCC TAC GGA GGC AGC AG and Univ516R: GTD TTA CCG CGG CKG CTG RCA) primers targeting the V2-V3 region of the 16S rRNA gene. Two standards prepared from *Thermococcus litoralis* and *Shewanella oneidensis* were used for archaeal and bacterial absolute quantification, respectively. Standard curves were prepared through the cloning method using pGEM(R)-T Easy Vector System II (Promega, USA) and JM109 Competent Cells (Promega). The plasmid was extracted with PureYield™ Plasmid Miniprep System (Promega) and quantified on a Quantus™ Fluorometer using the QuantiFluor(R) dsDNA System (Promega).

### 2.8 Coulombic efficiency (CE)

For all produced and consumed compounds, measured with the HPLC and IC, the coulombic efficiency (CE) was calculated to evaluate the ratio of produced and consumed electrons according to:


$$CE\;\left(\%\right)=\frac{F\cdot n_{e}\cdot \Delta \left[P\right]\cdot V_{\mathrm{catholyte}}}{\int_{t_{0}}^{t}i\left(t\right)\cdot dt}\cdot 100$$

where F is the Faraday constant, ne is the number of moles of electrons present per mole of product created [mol], *Δ*[P] is the variation in product concentration between t0 and t [mol L-1], and V is the volume of the catholyte [L]. The integration of current over time was approximated with the current difference between consecutive timepoints according to


$$\sum \left(I\cdot \left(t_{n+1}-t_{n}\right)\right)$$

with I being the current [A] and t being the time [s]. The total coulombic efficiency was calculated as a sum of the proportional coulombic efficiencies.

### 2.9 DNA sequencing

Sonicated biofilms previously prepared for qPCR were directly used in PCR without extraction to prevent DNA loss with 5 μL of sample or diluted sample in a 50 μL PCR mix. The V4 region of the 16S rRNA gene was amplified using the universal primers 515F (5′-GTG CCA GCM GCC GCG GTA A-3′) and 806R (5′-GGA CTA CNN GGG TAT CTA AT-3′), the archaeal primers Arc 344F (5’-ACGGGGYGCAGCAGGCGCGA-3′) or Arc519F (5’-CAGCMGCCGCGGTAA-3′) and Arch806R (5’-GGACTACVSGGGTATCTAAT-3′) with Taq&Load MasterMix (Promega) in triplicates and pooled together. PCR reactions were carried out using a thermal cycler (iCycler, Bio-Rad) with the following conditions: initial denaturation at 94°C for 3 min followed by 30 cycles of denaturation at 94°C for 30 s, primers annealing at 50°C for 30 s and extension at 72°C for 90 s, followed by a final extension step of 5 min at 72°C. Controls with water were performed to check for analytical contaminations. The amplified gene regions were sequenced on an Illumina MiSeq 2,500 platform (GeT-PlaGe, France) to generate paired-end 250 bp reads. The amplicon sequencing datasets generated for this study can be found in the NCBI SRA database under the accession number: PRJNA1187372.

### 2.10 Bioinformatic analyses

The amplicon sequencing data was processed using the DADA2 software package (version 1.22.0) (21) in R (version 4.1.2) and following the steps in the online tutorial^1^.

During the initial filtering step, the first 17 and 21 nucleotides of the forward and reverse reads, respectively, were trimmed to remove the primers. The last 20 nucleotides of all reads were trimmed as their sequencing qualities were lower. Three expected errors in each read were tolerated to increase the number of reads retained after filtering.

Samples were normalized to 100,000 reads for beta-diversity analysis. Samples with less than 100,000 reads were not represented. For representation, % of abundance where projected on the bacteria versus archaea proportions based on the qPCR values.

The Multi Factorial Analysis (MFA) and Non-Metric Multidimensional Scaling (NMDS) were also performed on R 4.1.2 with the RStudio interface. The FactoMineR 2.6 (Lê et al., 2008) and factoextra 1.0.7 packages were used for the MFA. Quantitative variables were standardized to unit variance. For the NMDS, relative abundances at the genus level and the phyloseq package were used.

## 3 Results

### 3.1 Bioelectrochemical performances of 60 reactors

**Figure 2:**
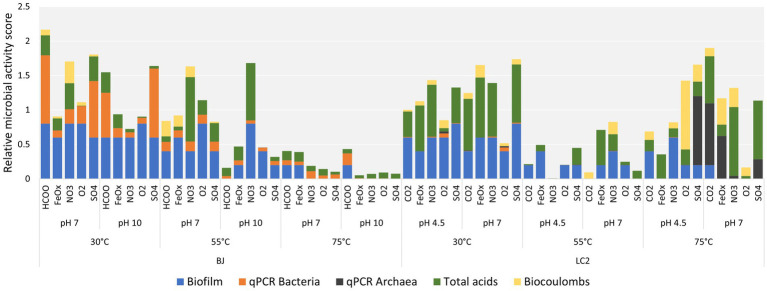
Relative microbial activity of all reactors. The relative microbial activity scores are cumulative scores obtained from five indicators of microbial activity: biofilm, bacteria number (qPCR), archaea number (qPCR), organic acid production and biocoulombs. Biocoulombs correspond to coulombs from current densities values higher (in absolute values) than the current densities reached after exactly 1 h of experiment (abiotic baseline). For each category, data was normalized from 0 to 1, with 1 corresponding to the maximum values across all reactors for continuous values (qPCR, biocoulombs and acids), or the maximum of the scale for discrete values (biofilms, where the maximum was defined as 5 for full coverage of the electrode, but not reached in our experiments). For each reactor, the total height of the bar corresponds to its cumulative, relative microbial activity score. By focusing on the height of one color only, each indicator (e.g., biofilm) can be compared across reactors independently from the other indicators.

Each reactor was designated by its inoculum (BJ: ‘Bain des Japonais’ site in Prony Bay or LC2: La Calcara site in Panarea), temperature, pH, and electron acceptor. ‘FeOx’, ‘NO3’, ‘O2’, and ‘SO4’ refer to iron oxide, nitrate, dioxygen, and a sulfate/thiosulfate mixture, respectively. ‘HCOO’ and ‘CO2’ reactors have no additional electron acceptor beyond the carbon source (formate for BJ and carbon dioxide for LC2). As reminder, formate was used on BJ experiment to represent the carbon source in the environmental conditions where CO_2_ precipitates with the high pH hydrothermal fluid. Figure 2 shows the relative microbial activity of all reactors based on biofilm formation, qPCR, organic acid production, and electricity consumption. All biofilms and more details on how biofilm scores were obtained can be found in the Supplementary material. Generally, cells were observed for all temperatures and pH values, although more biofilm was obtained at a lower temperature of 30°C. Different cell morphologies were observed (Supplementary Figure S1). Bacterial 16S gene copies were consistently higher in BJ reactors. This could be explained by the presence of formate (20 mM) in BJ reactors. While it was provided as a carbon source, formate can also serve as an electron donor, meaning that microorganisms are not limited to cathode oxidation. A maximum of 3.40 × 10^10^ 16S gene copies/cm^2^ was measured for bacteria and was found in the BJ-30°C-pH10-SO4 reactor. For archaea, the maximum was 9.93 × 10^10^ 16S gene copies/cm^2^ (LC2-75°C-pH 4.5-SO4).

For the BJ inoculum, the most microbially active reactors were found at 30°C and 55°C, including some alkaline ones. Low activity scores were found in all the BJ-75°C reactors, suggesting that hyperthermophiles were poorly enriched. Among BJ-30°C-pH10 reactors (hydrothermal fluid conditions), the HCOO and SO4 reactors yielded the best scores, primarily because of higher amounts of bacterial 16S rRNA gene copies. However, they did not perform better than their neutrophilic counterparts (BJ-30°C-pH7).

For the LC2 inoculum, the effect of the temperature was also striking, albeit different than for BJ, with almost all the 30°C and 75°C reactors performing better than equivalent reactors at 55°C. Interestingly, the high microbial activity scores at 30°C were due to biofilms and organic acid production, while the high scores obtained at 75°C (LC2 hydrothermal fluid conditions) were also due to the important contribution of the archaeal 16S gene copies number and biocoulombs factors. This suggests that hyperthermophilic archaea have been enriched in LC2-75°C reactors.

### 3.2 Multiple factor analysis of data from all reactors

**Figure 3:**
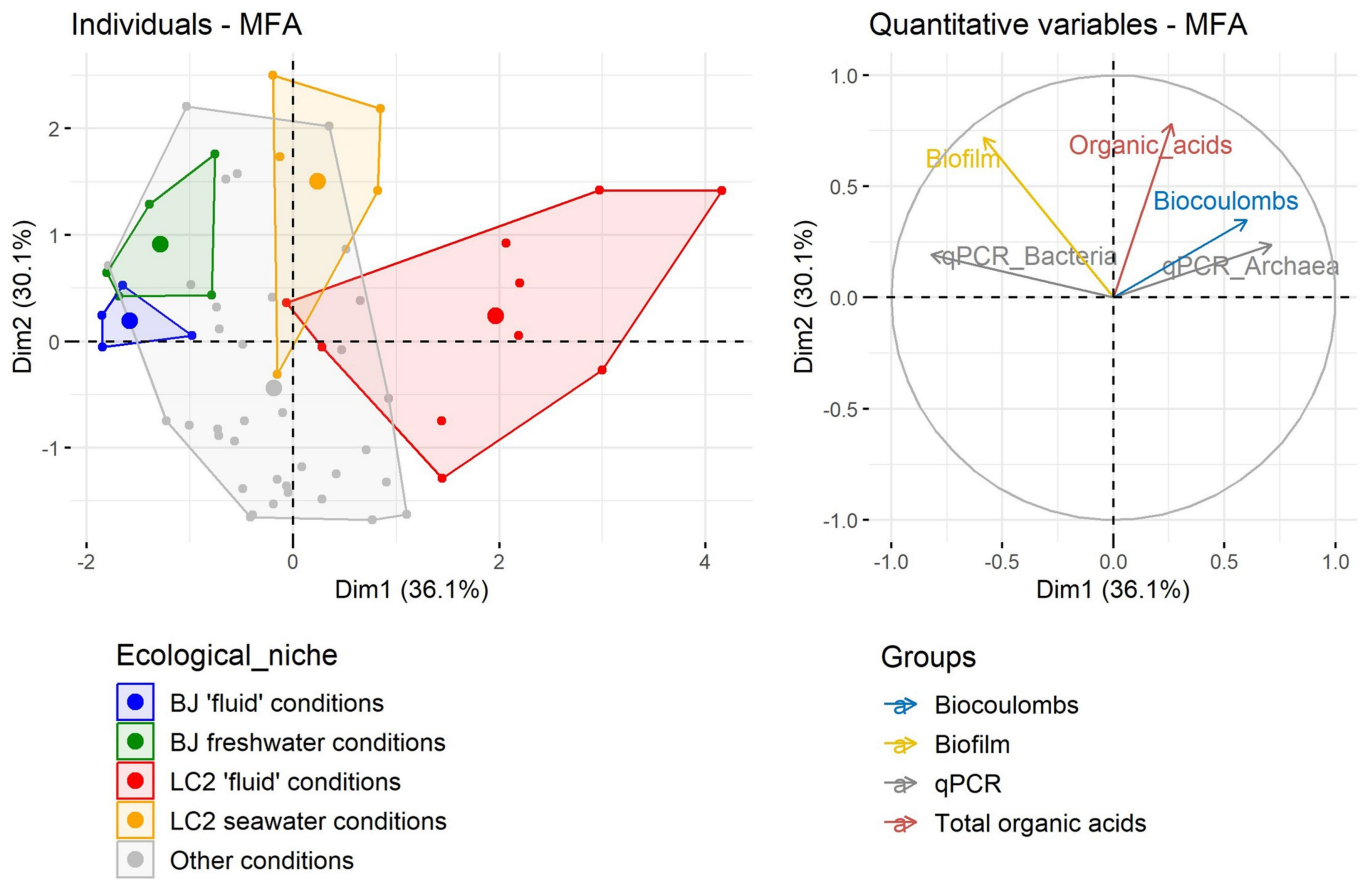
Multiple Factor Analysis (MFA) using growth data and current data from all 60 reactors.

In order to better summarize the reactor performance data and to compare ecological niches (temperatures and pHs), a Multiple Factor Analysis (MFA) was conducted on the data (Figure 3). The most important variations across reactors were due to qPCR and biocoulombs, correlated with the first dimension (38.5% of the variance). The second dimension (30% of the variance) was mainly explained by the biofilms and organic acid production. The LC2 reactors in the respective hydrothermal conditions (75°C, pH 4.5 and 7) were the most diverging reactors on the first dimension, owing to their higher amounts of archaeal 16S rRNA gene copies and biocoulombs, and smaller amounts of bacterial 16S rRNA gene copies. The analysis also shows in the second dimension that more biofilm and organic acids were produced in BJ and LC2 reactors with non-extreme conditions (30°C, pH 7) compared with hydrothermal conditions. Moreover, reactors could not be clearly grouped together based on other variables such as electron acceptors, pH, temperature or microbial taxa (not shown).

### 3.3 Electricity consumption in all reactors

Reactors were operated for 6 days in batch mode with a graphite plate cathode polarized at −0.6 V vs. SHE and an anion exchange membrane to separate the cathode and the anode. The duration of the experiment was chosen to enrich potential electroautotrophic first colonizers and limit subsequent heterotrophic growth relying on primary production. In addition, short experiments might prevent the fast degradation of fast-growing hyperthermophilic biofilms.

**Figure 4:**
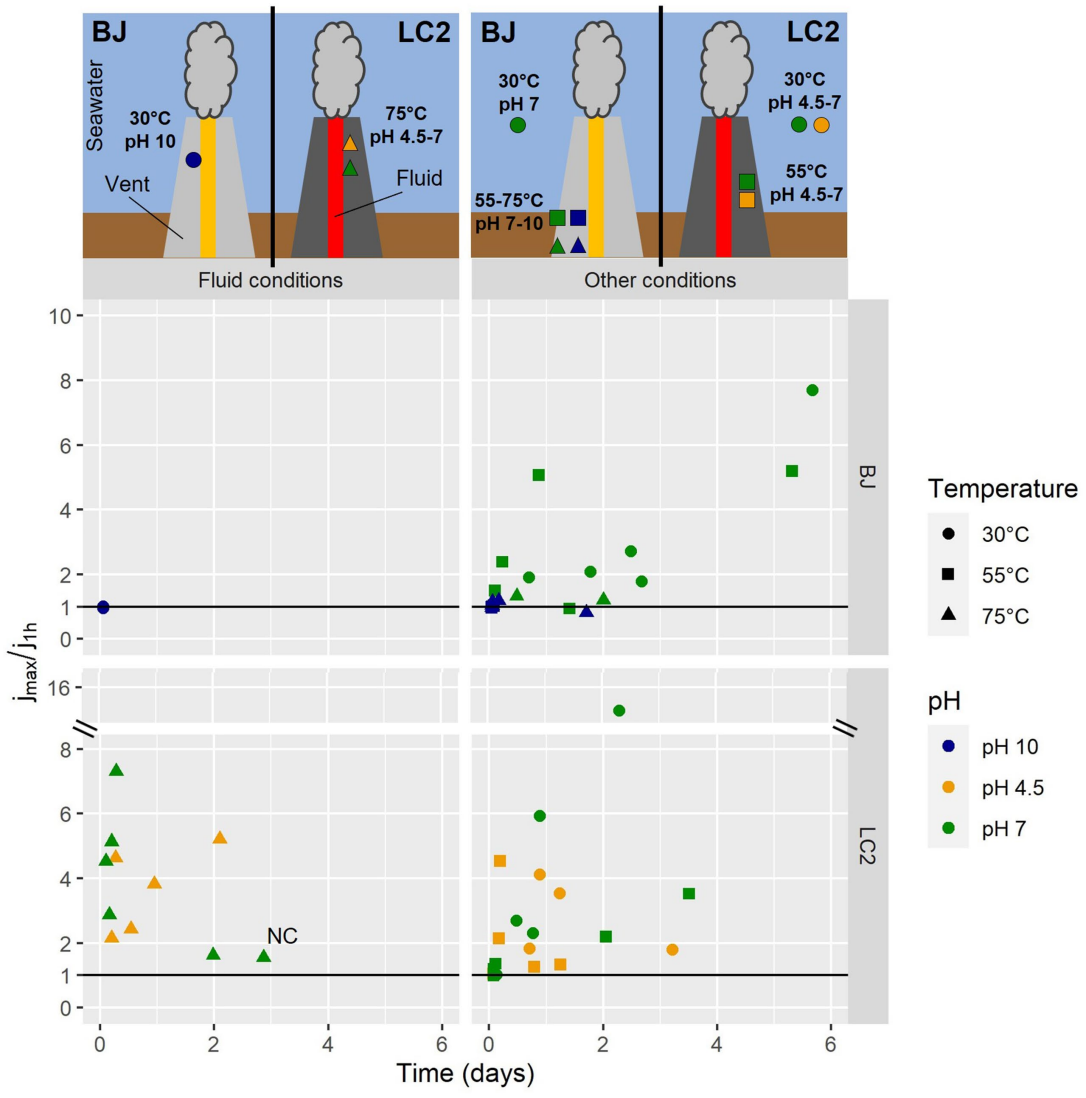
Current consumption in all 60 reactors. Each dot represents one reactor. j_1h_ is the current density reached after the first hour of experiment post-inoculation. j_max_ is the maximum current density observed beyond the first hour of the experiment. On the horizontal axis is the time it took to reach j_max_ in the reactor after the start of the experiment. The reactors were operated for 6 days. NC = negative control: abiotic (not inoculated) reactor and with a mix of all electron acceptors except O_2_. Above the graph, two schematics illustrate approximately the ecological niches (pH, temperature) of the ‘fluid conditions’ and ‘other conditions’ represented by the reactors, with respect to the distance from the hydrothermal fluid. Note that LC2 site is a vent without any chimney structure.

Figure 4 shows the maximum current densities in all reactors, expressed as a fold-increase compared to the abiotic baseline current. In BJ reactors operated in hydrothermal fluid conditions (30°C and pH 10), the current densities never reached values above the initial current densities (j_max_/j_1h_ ≤ 1). Remarkably, the same was observed for all BJ alkaline reactors, suggesting a poor ability to oxidize the cathode by alkaliphilic microorganisms in those reactors. The presence of biofilms in those reactors (Figure 2) suggests that the polarized graphite plates were colonized for other reasons than electrotrophic growth (electrons for energy), such as a low redox potential or physical support for biofilm formation. As previously said, the cathode in BJ reactors was a facultative electron donor, as formate (carbon source) could also have provided electrons for metabolism (i.e., methylotrophic growth).

For LC2 reactors with hydrothermal fluid conditions, maximum current densities were all higher than initial current densities. On average, a 6-fold increase was measured, while it was 1.6 for the abiotic control, suggesting that the current increases in inoculated reactors is mostly of microbiological origin. For 8 out of 10 reactors, these maximum values were reached during the first day of the experiment, suggesting that the exponential phase of growth was reached rapidly after the start of the experiment. In other conditions (Figure 4, right), e.g., non-extreme conditions, various maximum current density values were obtained (up to a 17-fold increase), usually during the first half of the experiment. These results suggest that hyperthermophiles in LC2 reactors were able to grow electrotrophically, consuming more electrons than mesophiles (Figures 2–4).

### 3.4 Metabolic activity of microorganisms

**Figure 5:**
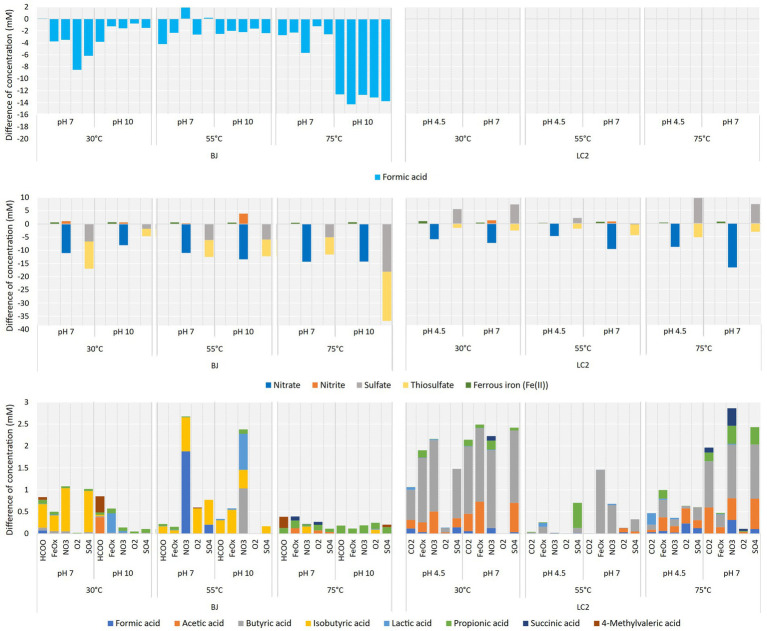
**(Top)** Formic acid consumption in BJ reactors. Differences in concentration between the start and the end of the experiment (6 days). Middle panel: Electron acceptors utilization. Differences in concentrations of nitrate, nitrite, sulfate and thiosulfate between the start and the end of the experiment in corresponding reactors. Initial concentrations were at 20 mM. **(Bottom)** Organic acid production. Differences in concentrations of 8 organic acids between the start and the end of the experiment in all reactors.

The LC2 reactors all had CO_2_ (20%) provided as a carbon source, while it was formate (20 mM) for the BJ reactors to reflect environmental conditions. Formate concentration decreased in most BJ reactors (Figure 5, top). The most significant differences were found in the five BJ-75°C-pH10 reactors (between 12 and 14 mM). Because values are very similar in these 5 reactors, and because all other indicators of growth are low (Figure 2), formate was likely transformed abiotically in those reactors with the help of catalyst provided by the inoculum. Indeed, the endothermic decarboxylation of formate is more favorable at high temperature and in its deprotonated form, dominating at pH 10. Formate anions might also have been transported from the catholyte to the anolyte through the anion exchange membrane. However, this should also have happened in the other BJ reactors. At lower temperatures, formate consumption was more varied, suggesting a biological process. Interestingly, in BJ-30°C-pH10 reactors (hydrothermal fluid conditions), formate was most consumed in the absence of an additional electron acceptor (BJ-30°C-pH10-HCOO, 3.8 mM). In contrast, at 30°C and pH 7 (favoring non-extremophiles), formate was only consumed with additional electron acceptors, with a maximum consumption of 8.5 mM with dioxygen.

The concentrations of nitrate, nitrite, sulfate, and thiosulfate were monitored by ion chromatography (Figure 5, middle). Nitrate was consumed in all BJ-and LC2-NO3 reactors (12 reactors), from 4.8 mM (LC2-55°C-pH4.5-NO3) to a maximum of 16.7 mM (LC2-75°C-pH7-NO3). Nitrite, a product of microbial nitrate reduction, was detected in 6 of those reactors (up to 3.9 mM produced in BJ-55°C-pH10-NO3). Thiosulfate was consumed in all LC2-and BJ-SO4 reactors (between 1.6 and 18.6 mM). Whereas sulfate concentrations decreased in all BJ-SO4 reactors, they mainly increased in LC2-SO4 reactors. As sulfate can be a product of microbial thiosulfate oxidation, it is possible that thiosulfate was used as an electron donor rather than an electron acceptor in these reactors. In general, these results did not correlate well with the rest of the data (Figures 2, 4), i.e., higher consumptions did not correlate with higher gene copy numbers and current consumptions. As anion exchange membranes were used, it cannot be excluded that some electron acceptors moved between the catholyte and the anolyte because of the potential difference. Anions are expected to move from the catholyte to the anode, contributing to the concentration decreases seen in our data. Additionally, iron reduction was detected in all BJ-and LC2-FeOx reactors (see also Supplementary Table S2), up to 0.9 mM, which represents around 30% of Fe(III) initially available. This ratio is expected from the stoichiometric conversion of the iron(III) oxide into magnetite (Fe^2+^)_2_(Fe^3+^)O_4_, a typical product of microbial iron oxide reduction (Lovley et al., 1987). Correspondingly, particles in these reactors generally turned darker and magnetic.

Organic acids were produced in most of the reactors (Figure 5, bottom). The highest productions were measured in LC2 reactors (30°C and 75°C), as shown previously (Figure 2), except for 2 BJ-55°C reactors. Up to 2.9 mM of organic acids were produced after 6 days (LC2-75°C-pH7-NO3). Isobutyric acid was the dominant acid detected in BJ reactors, but various profiles were found. In BJ-30°C-pH10 reactors (fluid conditions), mostly lactic, acetic, propionic, and 4-methylvaleric acids were detected. In most LC2 reactors, butyric acid was the dominant acid, and smaller amounts of mainly acetic, propionic, succinic, and formic acids were detected.

These organic acids may have been produced via Microbial Electrosynthesis, i.e., the reduction of inorganic carbon (CO_2_ or formate) into organic compounds by electroautotrophic metabolisms. However, although conditions in the reactors were defined to favor those metabolisms, low concentrations of yeast extract (0.1 and 0.2 g/L in BJ and LC2 reactors, respectively) were added to provide micronutrients, vitamins and essential amino acids to stimulate growth. It is possible that yeast extract was fermented in our reactors. Its consumption in reactors could not be directly measured, but the Chemical Oxygen Demand (COD) of the initial and final concentration was measured. The calculated COD of the produced organic acids was subtracted from the final measurement to define the COD from the remaining yeast extract or biomass (Supplementary Figure S6). Only 6 of the BJ reactors showed higher COD at the final time than at the initial condition, while most of the LC2 reactors showed a significant increase of COD, with between 4 to 12 g of COD in some reactors at 75°C (CO_2_ and SO_4_ at both pH and NO_3_ and O_2_ at pH 7). These COD increases suggest a carbon fixation through microbial activity.

**Figure 1:**
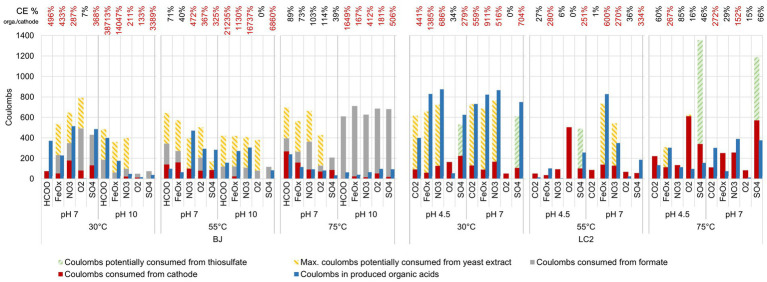
Potential electron donors for microorganisms in our reactors. Coulombs distribution in all reactors over the 6-day experiments, based on the electrochemical current and the consumption of formate and thiosulfate. Thiosulfate oxidation was assumed in the reactors which showed concomitant and almost stoichiometric thiosulfate consumption and sulfate production. Coulomb values from the potential consumption of yeast extract are calculated from the final COD value (see Materials and methods for more details and Supplementary Figure S6 for all COD values). The coulombic efficiency (CE) on top of the barplot represents the percentage of coulombs present in the organic acids compared to the coulombs provided by the electrode. A percentage superior to 100% (in red) indicates the use of an additional electron donor to explain the production of the organic acids.

To identify the electron donor used and the potential electroautotrophic communities, a comparison of the coulombs from the four potential electron donors (the cathode, formate (BJ reactors only), yeast extract, and thiosulfate) and the coulombs in the organic acids produced is shown in Figure 1. Thiosulfate was considered as a potential electron donor only in the 5 LC2 reactors where thiosulfate consumption was concomitant with sulfate production (Figure 4B). In 8 of the BJ reactors, the organic acid production could be explained by the coulombs provided by the cathode alone, especially at 75°C pH 7. In the other cases, the organic acid production can only be explained by a combination of the 3 electron donors: cathode, yeast extract, and formate. This suggests an electro-fermentation process, meaning that the cathode did not serve as main electron donor (as for electrotrophy), but provided additional electrons during fermentation, possibly shifting the fermentation to more reduced products (Moscoviz et al., 2016). Similar observations can be made for most LC2 bioreactors at 30°C and 55°C. However, in the bioreactors LC2 75°C, the organic acid production (and additional COD) can only be explained by the coulombs provided by the cathode, suggesting an electroautotrophic metabolism.

### 3.5 Microbial diversity in reactors and potential electrotrophs

16S metabarcoding using universal prokaryotic primers was performed to identify the microbial communities enriched on the electrodes. Archaea were not detected in the amplicon sequencing data (< 0.01%) despite some reactors having mostly archaeal 16S gene copies according to qPCR. No amplicons could be obtained with archaeal specific primers (Arch344-Arch806) on any conditions without reaching non-specific conditions (amplification of PCR negative control and too low hybridization temperature). *Thermococcus*, a hyperthermophilic archaeal genus, was the only archaeal taxa detected in a second attempt of 16S metabarcoding analysis with Archaea specific primers (Arc519F-Arc806R) performed on 5 reactors with some of the highest archaeal 16S gene copy numbers with archaeal-specific primers (data not shown). These results were confirmed by qPCR with *Thermococcales* specific primers (see Supplementary Table S2) but did not reach the qPCR archaeal values, suggesting the presence of other archaeal groups. Tests with other specific primers for different archaeal orders (*Crenarchaeota*, *Archaeoglobales*, methanogens) did not show significative amplification. Several reasons could explain the different results obtained with 16S metabarcoding and qPCR, including a bias of the 16S universal primers favoring bacterial 16S DNA amplification, a low coverage of the archaeal taxa by these primers (56.4% coverage of Archaeal domain, 0% of *Thermococcales* order), or a fast degradation of 16S DNA of the fast-growing and -decaying hyperthermophilic archaea, not detected with the longer expected amplicons (462 bp) of metabarcoding primers but detected with the short qPCR amplicons (187 bp) (see Discussion for more details).

**Figure 6:**
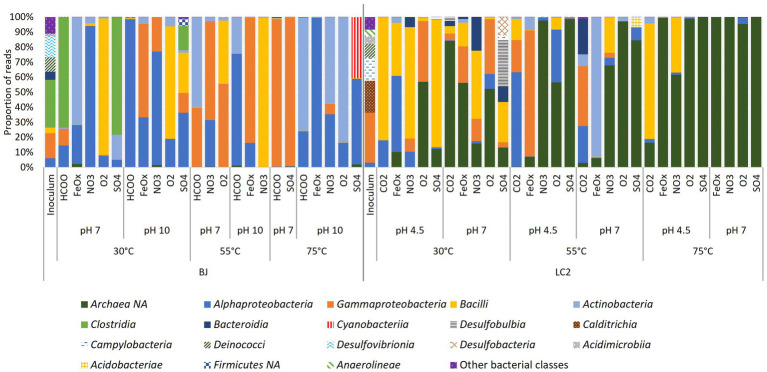
Detection frequency of prokaryotic classes in reactors. Amplicon sequencing reads (from 16S metabarcoding) were normalized to 100,000. Eight reactors with much less reads (500 to 45,000) than the others were excluded from this analysis. Archaea were not detected despite the use of universal primers and their dominance in some reactors according to qPCR results. The data was normalized by the proportion of bacteria and archaea according to qPCR. The legend includes classes with a minimum of 1% in a reactor. NA = non-assigned.

To account for the qPCR results, the amplicon sequencing data was normalized by the proportions of bacteria and archaea in the reactors according to qPCR results (Figure 6). Many LC2 reactors, especially at 75°C, were dominated by archaea (dark green). Other reactors were dominated mostly by 4 bacterial classes: *Alpha-and Gamma*-*proteobacteria*, *Bacilli*, and *Actinobacteriota*. Several typically thermophilic classes were abundant in the inoculum but not in the reactors, including *Calditrichia*, *Campylobacteria*, and *Deinococci*. About 60% of reads in the inoculum belonged to bacterial phyla that were not detected in our enrichments. Archaea were not abundant in BJ reactors, even after normalization. The same bacterial classes were dominant in BJ, one of the main differences with LC2 reactors being the additional abundance of *Clostridia*. These results show that different communities were enriched across reactors.

**Figure 7:**
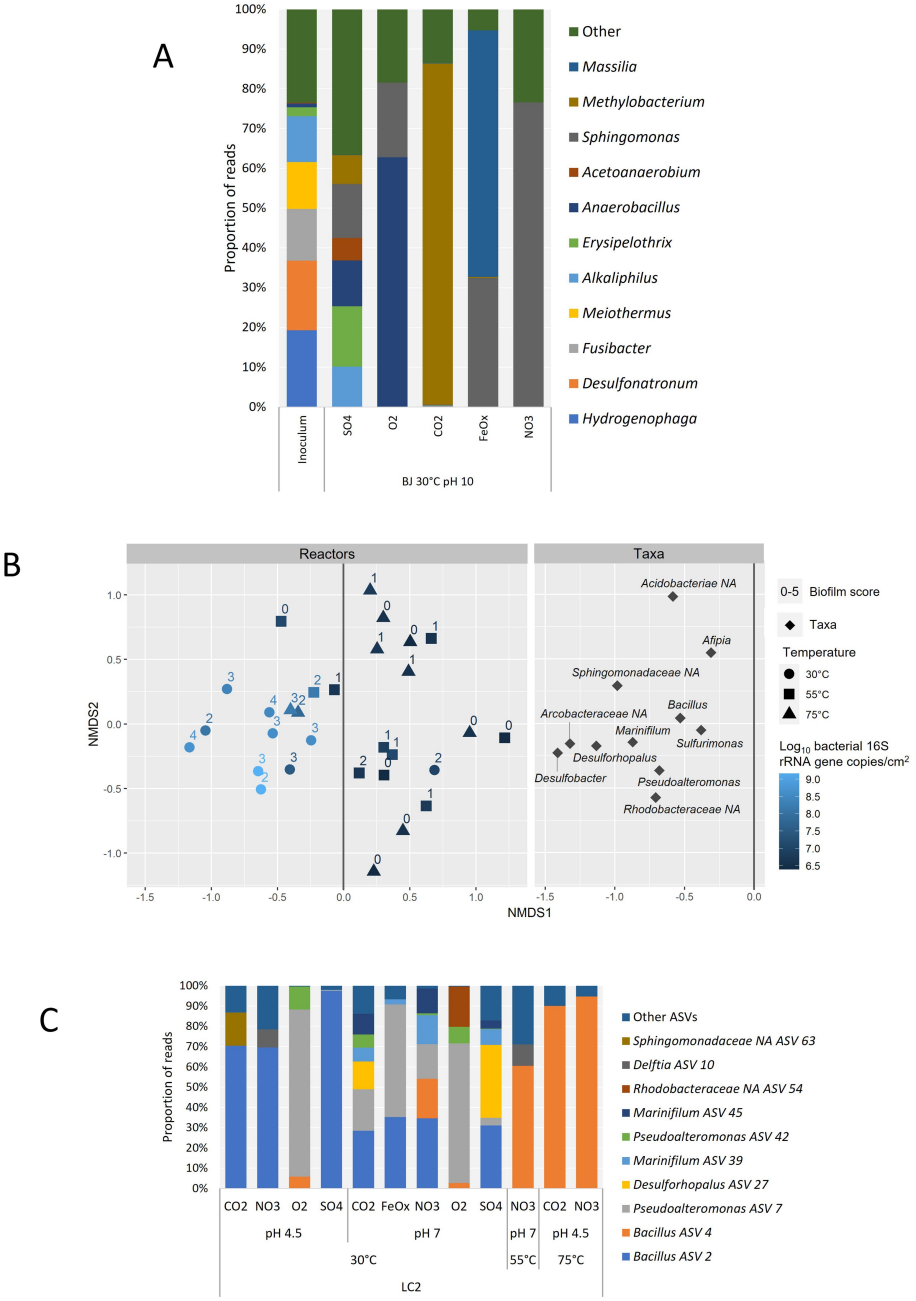
Microbial diversity at the genus level based on amplicon sequencing data only. **(A)** Detection frequency of prokaryotic genera in BJ reactors operated in hydrothermal fluid-like conditions (30°C, pH 10) and comparison with the BJ inoculum. Only the 10 most abundant genera are shown for clarity. **(B)** Non-Metric Multidimensional Scaling (NMDS) biplot based on the relative abundances of genera (minimum 1%) in LC2 reactors and Bray-Curtis dissimilarities. Only the left side of the ‘Taxa’ plot, which correlates with higher growth in the reactors, is shown for clarity. For each reactor, the temperature, bacterial growth (qPCR) and biofilm score are indicated. **(C)** Detection frequency of prokaryotic ASVs in LC2 reactors corresponding to the left side of the NMDS plot above, and with a biofilm score of 2 or more (12 reactors). The genus affiliation is given for each ASV or, if none was obtained, the lowest identified taxonomic rank followed by “NA”: Non-Assigned at the genus level. Only the 10 most abundant ASVs are shown for clarity.

To identify potential indigenous electrotrophs, a focus was made on the reactors representing the hydrothermal fluid conditions. The microbial diversity at the genus level is shown in Figure 7A for BJ reactors at 30°C and pH 10 and compared with the inoculum. The most frequently detected genera in the reactors are mainly *Sphingomonas*, *Methylobacterium*, *Massilia*, and *Anaerobacillus*. Some of the main genera in the SO4 reactor are also found in the inoculum, namely *Alkaliphilus* and *Erysipelothrix* (Figure 7A). The two dominant genera in the inoculum are *Hydrogenophaga* and *Desulfonatronum*, and they were not detected in any of the BJ reactors.

In LC2 reactors, a correlation between biofilm score and bacterial 16S gene copy number was observed (Figure 7B). Indeed, a non-metric multidimensional scaling (NMDS) allowed to separate the reactors with the most growth from most of the others based only on their taxa compositions. In this group, all but one 30°C reactor were comprised, as well as two 75°C reactors and one 55°C reactor. These reactors were associated mainly with the following genera: *Desulfobacter*, *Desulforhopalus*, *Marinifilum*, *Pseudoalteromonas*, *Bacillus*, and *Sulfurimonas*. The three other unidentified genera on the graph represented 3 ASVs from the *Arcobacteraceae*, *Rhodobacteraceae*, and *Sphingomonadaceae* families. The genus composition in the LC2 inoculum was unlike any of the LC2 reactors (Supplementary Figure S3). *Sulfurimonas* was the only genus detected both in the reactors and in the inoculum (2.1% of reads). *Bacillus* was the most abundant genus identified in the reactors with the most growth (Figure 7C). The *Bacillus* ASV 2 was abundant in all 30°C reactors that did not contain oxygen, suggesting an anaerobic metabolism independent of exogenous electron acceptors, i.e., fermentation. The *Bacillus* ASV 4 dominated the three higher-temperature reactors in this group (LC2-55°C-pH7-NO3, 58.3% of reads; LC2-75°C-pH4.5-CO2, 86.6%; and LC2-75°C-pH4.5-NO3, 92.0%), and was also present (> 0.1% of reads) in three 30°C reactors, including the two aerobic reactors (5.7% at pH 4.5; 2.6% at pH 7) and one with nitrate as electron acceptor (LC2-30°C-pH7-NO3, 18.2%), overall suggesting that it is a thermophile capable of using dioxygen and nitrate as electron acceptors. Besides *Bacillus*, the most abundant genera were *Pseudoalteromonas*, *Desulforhopalus* and *Marinifilum*, each of them enriched in multiple 30°C reactors. This specificity of biodiversity in BJ and LC2 enrichments compared to their inoculums, obvious at the phylum level (LC2) or genus level (BJ), indicates that the culture conditions effectively selected the growth of adapted genera.

## 4 Discussion

### 4.1 Metabolisms in the Prony hydrothermal field and BJ reactors

Hydrothermal-like BJ reactors had some of the most developed biofilms and the highest 16S rRNA gene copy numbers. However, the current consumption in these five reactors never increased during the 6 days of cultivation (in contrast to most neutral and acidic reactors), and the cathodic coulombs were too low to account alone for the organic acids produced. As formate, nitrate, sulfate, and thiosulfate were consumed and fermentation products were detected, growth was probably supported by methylotrophy and fermentation. Many of the taxa abundantly detected in BJ-30°C-pH10 reactors have been reported to use formate for growth, including *Methylobacterium* (Ardley and Green, 2023), *Sphingomonas* (Boden et al., 2008), *Bradyrhizobium* (Manian and O’Gara, 1982), and *Methylocella* (Dedysh and Dunfield, 2016). Curiously, all of them are known as strict aerobes but were enriched in aerobic and anaerobic reactors. Typical aerobic methylotrophic bacteria (metabolizing C1 compounds) are, in fact, also detected in anaerobic environments (Chistoserdova and Kalyuzhnaya, 2018). In some reactors, such as BJ-55-7-NO3 and BJ-55-7-SO4, the increase of COD coupled with the necessity to combine coulombs from yeast extract and cathode to explain the organic acid production suggest an electro-fermentation metabolism. A cathodic electro-fermentation is a microbial fermentation whereby the cathode is used as an additional electron source, allowing a shift towards more reduced metabolic products (Moscoviz et al., 2016). In these bioreactors, *Sphingomonas* and *Stenotrophomonas* species were dominant. *Sphingomonas* species have been previously enriched on biocathode (Nájera et al., 2020), while *Stenotrophomonas* have been described as electrode respiring (Venkidusamy and Megharaj, 2016). Because the cathode either was not used, or only provided part of the electrons necessary for the formation of the detected organic acids, none of these conditions seem to have enriched pure electrotrophs (cathodic electrons as sole electron donor). Nevertheless, some strains seem to be able to use the cathode as an additional energy source for electro-fermentation. Obtaining pure cultures would be necessary to confirm that these taxa grew using the electrode as secondary energy source.

The dominant genera in the BJ inoculum, *Hydrogenophaga* (*Gammaproteobacteria*) and *Desulfonatronum* (*Desulfobacterota*), are hydrogenotrophs typically found in alkaline hydrothermal environments (Popall et al., 2023). Indeed, hydrogen (H_2_) derived from the serpentinization reaction is the main electron donor for these communities (Quéméneur et al., 2023). Despite hydrogenotrophs being capable of electrotrophic growth through direct or mediated (H_2_) cathode oxidation (Gupta et al., 2020), these taxa were not enriched in our reactors. As the potential of the Hydrogen Evolution Reaction (HER) on the cathode depends on pH and temperature, it is necessary to evaluate its feasibility in our experimental conditions. Assuming H_2_ threshold concentrations reported in the literature for aerobic respiration, denitrification, iron reduction, sulfate reduction, and methanogenesis (Karadagli et al., 2023), the redox potential of the HER was calculated (see Supplementary Table S1). At pH 4.5, the redox potential was the most positive, with values ranging from −0.038 V (vs SHE) at 30°C to −0.104 V at 75°C. At pH 7, the potentials ranged from −0.188 V at 30°C to −0.277 V at 75°C. Finally at pH 10, the potentials ranged from −0.369 at 30°C to −0.484 at 75°C. As expected, the driving force of the HER becomes less favorable at high pH, supporting the higher current observed at low pH. The increase of temperature seems, however, to increase the current, while the reaction should be less favorable according to the redox potential. Nevertheless, it is also known that increasing the temperature increases the reaction rates by reducing the activation energy required for the reaction. The potentials at pH 10 are very close to the cathode potential (−0.6 V). Considering overpotentials, it is unlikely that H_2_ was sufficiently produced to favor hydrogenotrophic microbial growth. Based on cathodic coulombs, a total of 0.47 mM of H_2_ could have been produced over 6 days in BJ-30°C-pH10 reactors, at most. At 55°C and 75°C, the maximum calculated productions were 0.50 mM and 1.10 mM, respectively. When these concentrations are compared with the initial concentrations of formate (10 mM) and yeast extract (0.1 g/L) directly available at the start of the experiments, it is reasonable to consider that methylotrophic or other organotrophic metabolisms were favored instead.

This work provides the first attempt to enrich cathode-oxidizing microorganisms from serpentinizing ecosystems. Overall, the dominant taxa in our alkaline hydrothermal reactors are minor constituents of the inoculum and do not appear to be pure electrotrophs, while potential electro-fermentative species might have been enriched. This may be explained by the lack of electrotrophs in these environments. Indeed, the hydrothermal chimneys found in Prony Bay and other serpentinite-hosted environments are made of carbonates (CaCO_3_) and brucite (Mg(OH)_2_), electrical insulators (Launay and Fontes, 1985; Kelley et al., 2005; Monnin et al., 2014). Contrary to magmatically hosted hydrothermal vents, the concentrations of dissolved metals in their hydrothermal fluid are very low (Kelley et al., 2005; Ludwig et al., 2006). Therefore, these communities are not expected to use natural electric currents propagated by the chimney walls or dissolved metals as sources of electrons. Alternatively, experimental conditions may have favored fast-growing organoheterotrophs, while the duration of the experiments (6 days) may have been too short for slower autotrophic growth.

### 4.2 Metabolisms in the Panarea hydrothermal system and in LC2 reactors

Previous studies indicated that sulfur-oxidizing bacteria are dominant at the Panarea vents and that the aerobic oxidation of sulfur is the most thermodynamically favorable metabolism in the conditions around the vents (Maugeri et al., 2009, 2010, 2013; Gugliandolo et al., 2015; Price et al., 2015). Sulfur-oxidizing bacteria are detected in the LC2 inoculum, including *Thioprofundum* (*Gammaproteobacteria*) (7.8%) and *Sulfurimonas* (*Campylobacterota*) (2.1%) (Supplementary Figure S3). However, the most enriched taxa in the LC2 reactors with the best growth (biofilms, qPCR) are generally described as organoheterotrophic, marine bacteria that have been isolated from non-hydrothermal habitats, such as seawater and marine sediments (Table 1). The two most abundant genera, *Bacillus* and *Pseudoalteromonas*, have been enriched or cultivated on cathodes before (Debuy et al., 2015; Wu et al., 2018). In the reactors dominated by *Bacillus* especially, rod-shaped cells are clearly observed on the surface of the electrode (Supplementary Figure S2), biotic current consumptions were observed and organic acids were produced. The coulombic efficiencies show that at low temperature, the cathode alone or yeast extract alone could not have provided enough electrons to explain these concentrations of organic acids, suggesting a combination of both through an electro-fermentation. Interestingly, butyrate, rather than acetate, was the dominant product, supporting that electro-fermentation occurred. Indeed, an increase of butyrate production over acetate has been observed before during the electro-fermentation of sucrose (Choi et al., 2012) and glucose (Paiano et al., 2019).

**Table 1:** Ecological and metabolic characteristics of the most frequently detected prokaryotic genera associated with the LC2 reactors which produced the most biofilm and had the highest bacterial 16S gene copy numbers (see Figure 6).

**Genus**	**Autotrophy**	**Metabolic type**	**Habitat**	**Electroactivity**	**References**	**Reactors with relative abundances > 1%**
*Bacillus*	Yes	Versatile	Ubiquitous including hydrothermal environments	Iron oxide reduction, anode reduction and cathode oxidation	Boone et al. (1995), Nimje et al. (2009), Logan and Vos (2015), and Wu et al. (2018)	LC2-30°C-pH4.5-SO4 (97%) LC2-75°C-pH4.5-NO3 (92%) LC2-75°C-pH4.5-CO2 (87%) and 9 others
*Desulfobacter*	Yes	Sulfate reduction	Marine, brackish or freshwater sediments, and activated sludge	Soluble iron reduction	Lovley et al. (1993) and Galushko and Kuever (2019a)	LC2-30°C-pH7-SO4 (14%)
*Desulforhopalus*	–	Sulfate reduction	Marine or brackish sediments	–	Galushko and Kuever (2019b)	LC2-30°C-pH7-SO4 (35%) LC2-30°C-pH7-CO2 (13%)
*Marinifilum*	–	Aerobic respiration	Seawater	–	Fu et al. (2018)	LC2-30°C-pH7-NO3 (12%) LC2-30°C-pH7-CO2 (10%) LC2-30°C-pH7-CO2 (4%)
*Pseudoalteromonas*	–	Aerobic respiration	Seawater and other marine environments	Cathode oxidation	Bowman and McMeekin (2015) and Debuy et al. (2015)	LC2-30°C-pH4.5-O2 (81%) LC2-30°C-pH7-O2 (68%) LC2-30°C-pH7-FeOx (55%) LC2-30°C-pH7-CO2 (19%) LC2-30°C-pH7-NO3 (16%) LC2-30°C-pH7-SO4 (4%)

The best grown LC2 reactors also include one 55°C reactor (LC2-75°C-pH4.5-NO3) and two 75°C reactors (LC2-75°C-pH4.5-NO3 and LC2-75°C-pH4.5-CO2), which are dominated by a *Bacillus* ASV showing 100% identity with *Bacillus licheniformis* (using BLAST). Rod-shaped cells of variable lengths were observed on the electrodes (Supplementary Figure S2). Some thermophilic strains of *B. licheniformis* have been isolated from hydrothermal vents of Panarea and other Aeolian islands before (Maugeri et al., 2002; Spanò et al., 2013). Although anaerobic growth and lithoautotrophic growth were not investigated, some of them were capable of nitrate reduction to nitrite (Spanò et al., 2013). As nitrate and biotic current were consumed in these reactors, this Bacillus ASV may represent a thermophilic, nitrate-reducing electrotroph.

Regarding archaea, an amplicon sequencing analysis using Archaea-specific primer could only obtain reads for one reactor, LC2-75°C-pH7-CO2, with 99% of reads belonging to *Thermococcus*, a well-described hyperthermophilic archaeal genus which has been firstly described from *T. celer* isolated from Vulcano (Zillig et al., 1983) and later isolated from Panarea (Price and Giovannelli, 2017; Gugliandolo and Maugeri, 2019). *Thermococcus* species are commonly heterotrophic and are capable of fermenting yeast extract, which is present in the medium (0.2 g/L). Cathodic biofilms of *Thermococcus* have been obtained recently with acetate as the carbon source (Popall et al., 2022). It is therefore possible that *Thermococcus* grew electroheterotrophically in one or several of the LC2 high-temperature reactors: using the cathode as electron donor and yeast extract as the carbon source. *Thermococcus* was indeed detected in LC2-75°C reactors by an additional qPCR analysis (Supplementary Table S2), while other archaeal phylogenetic groups abundantly detected in the LC2 inoculum or known to include hyperthermophiles were not.

According to qPCR results, many of the LC2 reactors were dominated by archaea. However, archaea were not detected with the 16S metabarcoding using universal primers, suggesting either that the NGS primers used were not optimal for this community, or that the 16S DNA fragment were partially degraded, due to the fast growth rate of hyperthermophiles and fast decay, allowing the 187 bp amplification of the qPCR but not the 460+ bp of the NGS amplicons. This hypothesis is supported by the absence of biofilm in hyperthermophilic condition after 6 days, while the current densities were the highest during the first day (Figure 4) and gradually declined over the 5 following days.

### 4.3 Biotechnological potential of electrotrophs from hydrothermal environments

Despite the potential benefits of thermophilic ME including faster microbial metabolisms, improved substrate solubility and mass transfer rates, and lower risks of contamination, very few thermophiles have been tested for ME (Dopson et al., 2016; Shrestha et al., 2018; Jourdin and Burdyny, 2021; Chaudhary et al., 2022). As they provide plenty of heat, metals, chemical energy and carbon dioxide to their microbial ecosystems, hydrothermal vents are ideal environments to look for novel thermophilic, autotrophic and electroactive biocatalysts for ME. To the best of our knowledge, shallow-sea hydrothermal vents have not been considered before in the search of such biocatalysts.

In our study, a thermophilic *Bacillus* was enriched from the hot, acidic Panarea hydrothermal sample. Although its metabolism and potential for electrosynthesis cannot be established yet, it is interesting to note that *Bacillus* is a major microorganism for the industrial production of enzymes and other compounds, due to their ability to secrete large extracellular quantities in various pH and temperature conditions (Harwood, 1989; Schallmey et al., 2004). In particular, the biotechnological potential of thermophilic *Bacillus*-related strains isolated from Panarea and Vulcano hydrothermal samples, has been described and investigated before (Nicolaus et al., 2000; Maugeri et al., 2002; Lentini et al., 2007; Gugliandolo et al., 2012; Spanò et al., 2013; Zammuto et al., 2022). These strains have been reported to produce exopolysaccharides, biosurfactants and enzymes at high temperatures and extreme pHs. With ME, these biocompounds could be produced from renewable electricity, instead of sugars or hydrogen. This approach would require the identification of autotrophic strains.

## 5 Conclusion

Bioelectrochemical enrichments were for the first time employed to assess the potential diversity of electrotrophs in shallow-sea hydrothermal vents. The results presented in this study suggest that electrotrophic microorganisms capable of feeding on electrical currents are present in volcanic shallow-sea hydrothermal vents. In particular, a low thermophilic diversity of potential electrotrophs was enriched, mainly composed of *Thermococcus* and *Bacillus*. Further research is necessary to characterize metabolisms, to confirm their biotechnological potential for Microbial Electrosynthesis, and to clarify whether respiratory pathways other than acetogenesis and methanogenesis can be exploited. In contrast, our alkaline reactors did not reveal the presence of electrotrophs in the serpentinite-hosted hydrothermal vent sample. This may be because serpentinite-hosted environments are not favorable for electrotrophs. However, our results point to the necessity of carefully defining the cultivation conditions for the enrichment of electrotrophs, as even low amounts of organic matter, in particular, may favor other metabolisms. Nevertheless, clear differences were observed for the two types of shallow-sea hydrothermal vents, providing valuable insights for microbial ecology/evolution and biotechnology.

## Data Availability

The datasets presented in this study can be found in online repositories. The names of the repository/repositories and accession number(s) can be found at: https://www.ncbi.nlm.nih.gov/, PRJNA1187372.

## Footnotes

^1^ https://benjjneb.github.io/dada2/tutorial.html
