# Supplementary material for: Enrichment of electrotrophic microorganisms from contrasting shallow-sea hydrothermal environments in bioelectrochemical reactors
Source: Front Microbiol. 2025 Feb 3;16:1539608. doi: 10.3389/fmicb.2025.1539608 (PMC11830809; doi:10.3389/fmicb.2025.1539608)
Supplement: Supplementary file 2 [file Data_Sheet_1.docx]

Supplementary Material

# Biofilms

At the end of the experiments (6 days). the cathodes of all 60 reactors were observed by fluorescence microscopy to check for biofilm formation. Cells were readily observed on the cathode of most reactors. Due to the possible electrodeposition of salts and minerals. the biofilms could not be quantified regarding cell numbers or electrode coverage but were given a visual score from 0 (no cells) to 5 (completely covered with cells). Two examples are shown in Supplementary Fig. 1 and described as follows. The BJ biofilm in Fig. 1A (left) was given a 3/5 score. The dominant cell shapes are long rods with what appear to be bulging. spherical terminal spores. The LC2 biofilm (right) had a 4/5 score. the maximum score among reactors. and was dominated by shorter and wider rod-shaped cells.


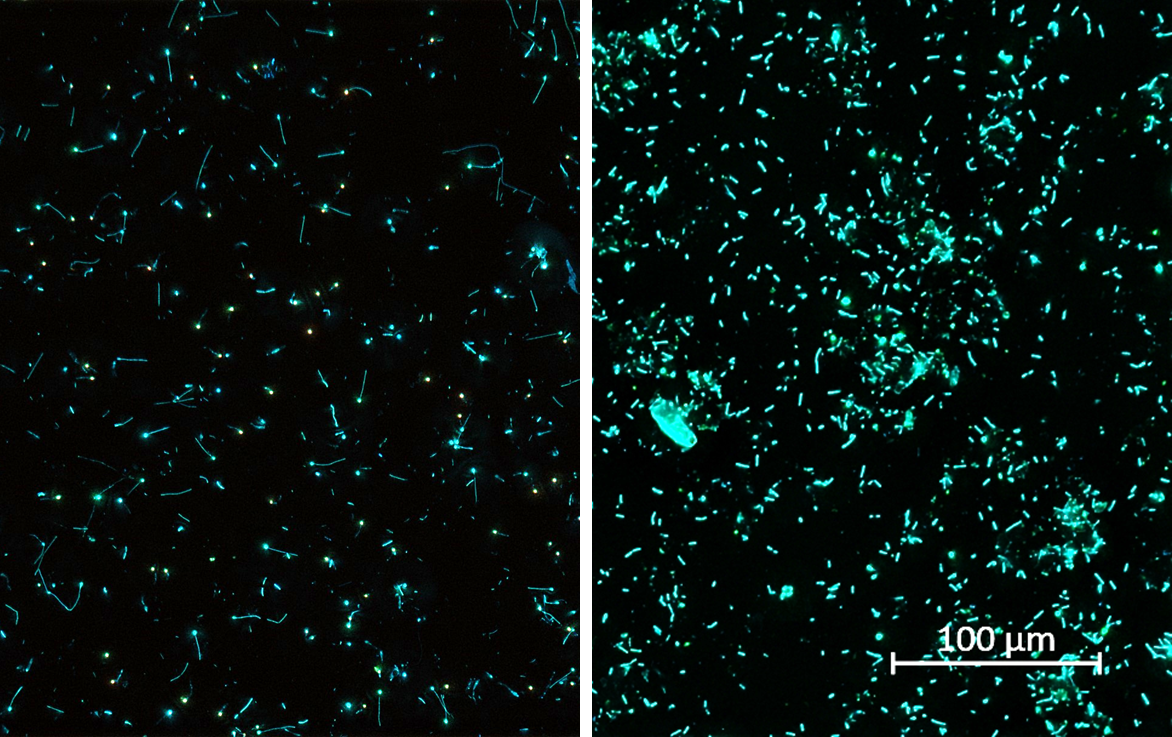


**Supplementary Figure 1.** Two examples of biofilm on graphite plate cathodes. Left: BJ-30°C-pH10-SO4. Score: 3/5. Right: LC2-30°C-pH4.5-SO4. Score: 4/5.

**Supplementary Figure 2.** (Below). Surfaces of graphite plate cathodes from all 60 reactors observed with a fluorescence microscope. For each cathode picture. the biofilm score is given in the top right corner. Two pictures were lost during processing with the microscope software.


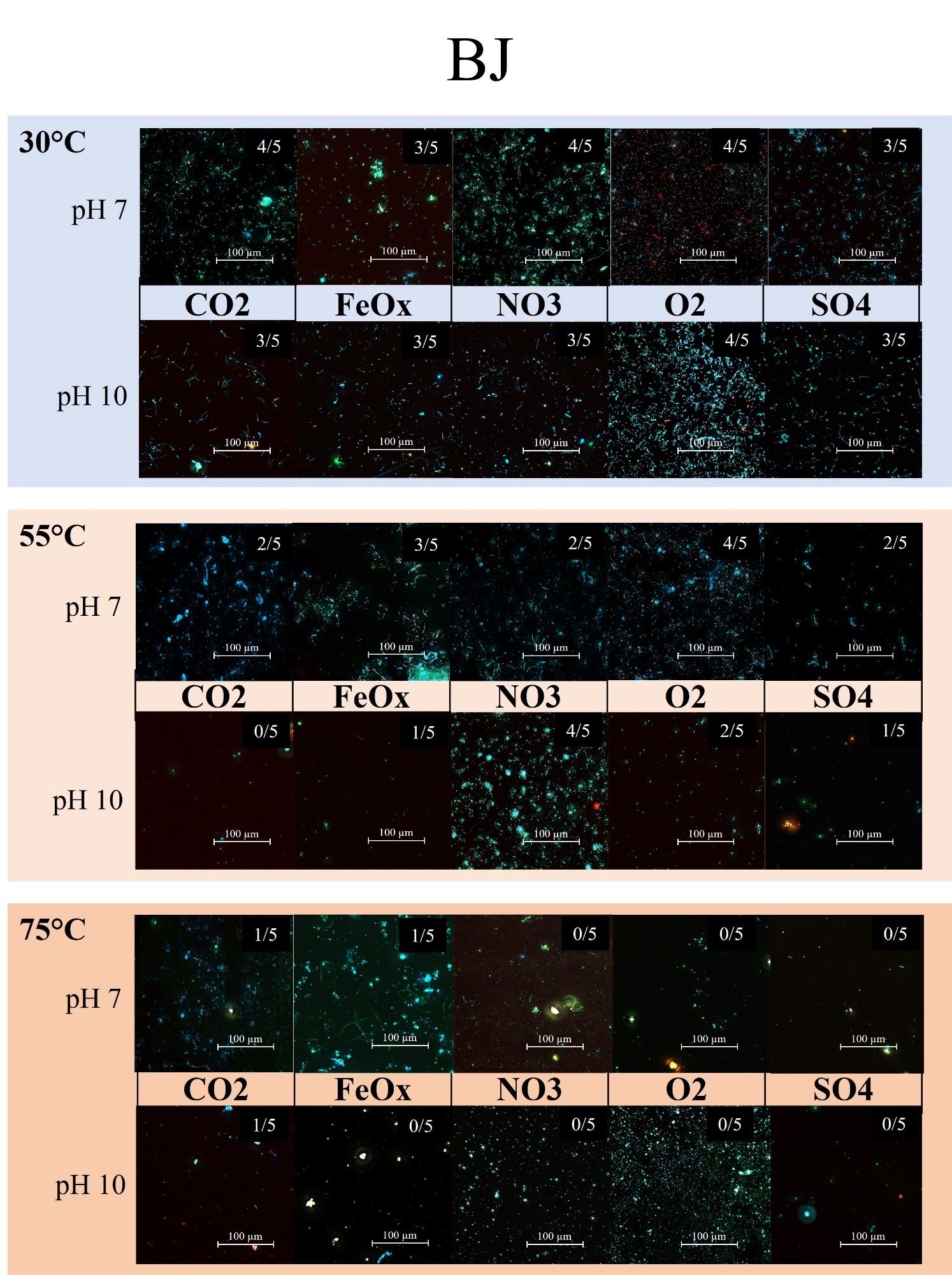

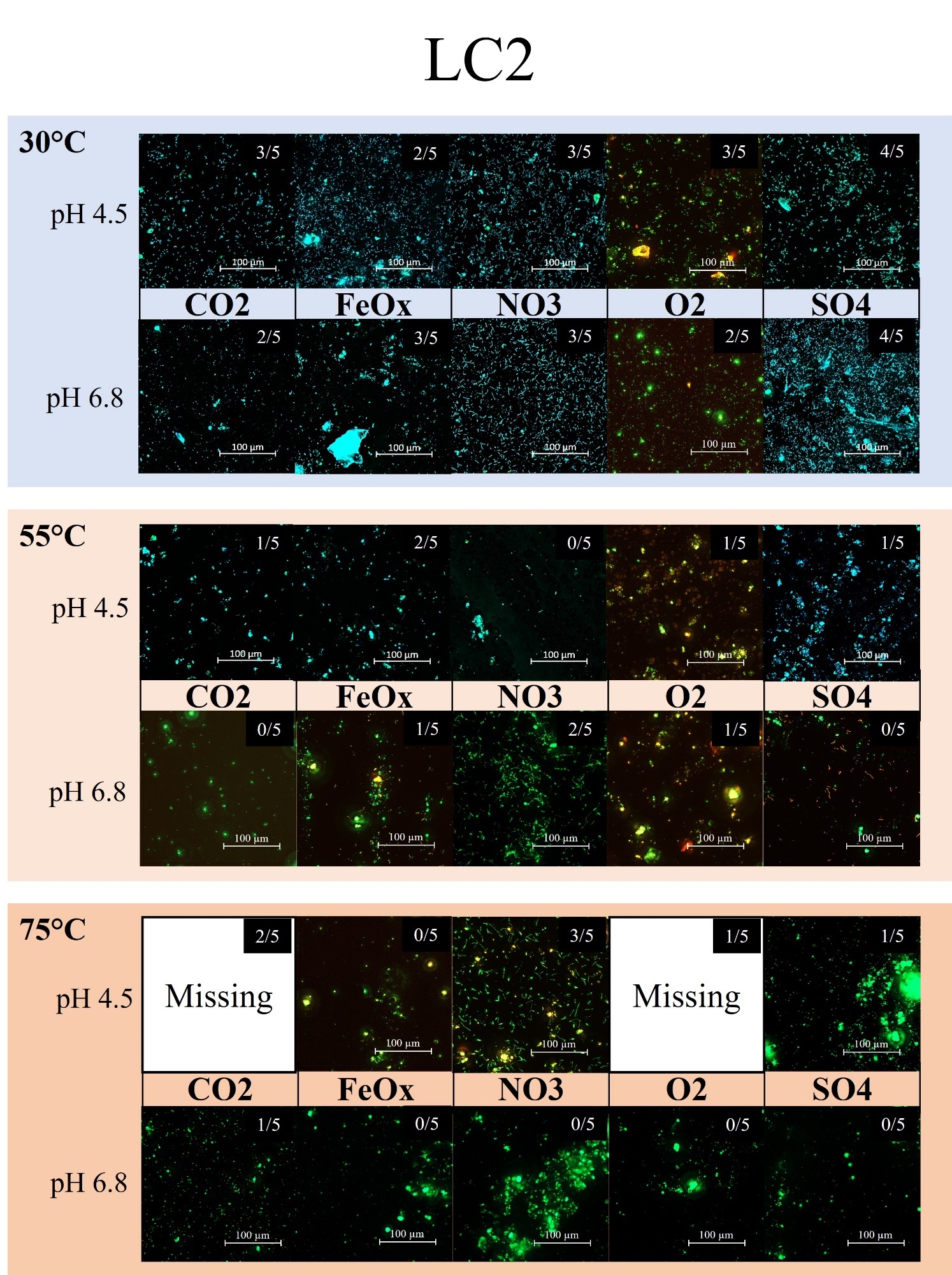


# Microbial diversity


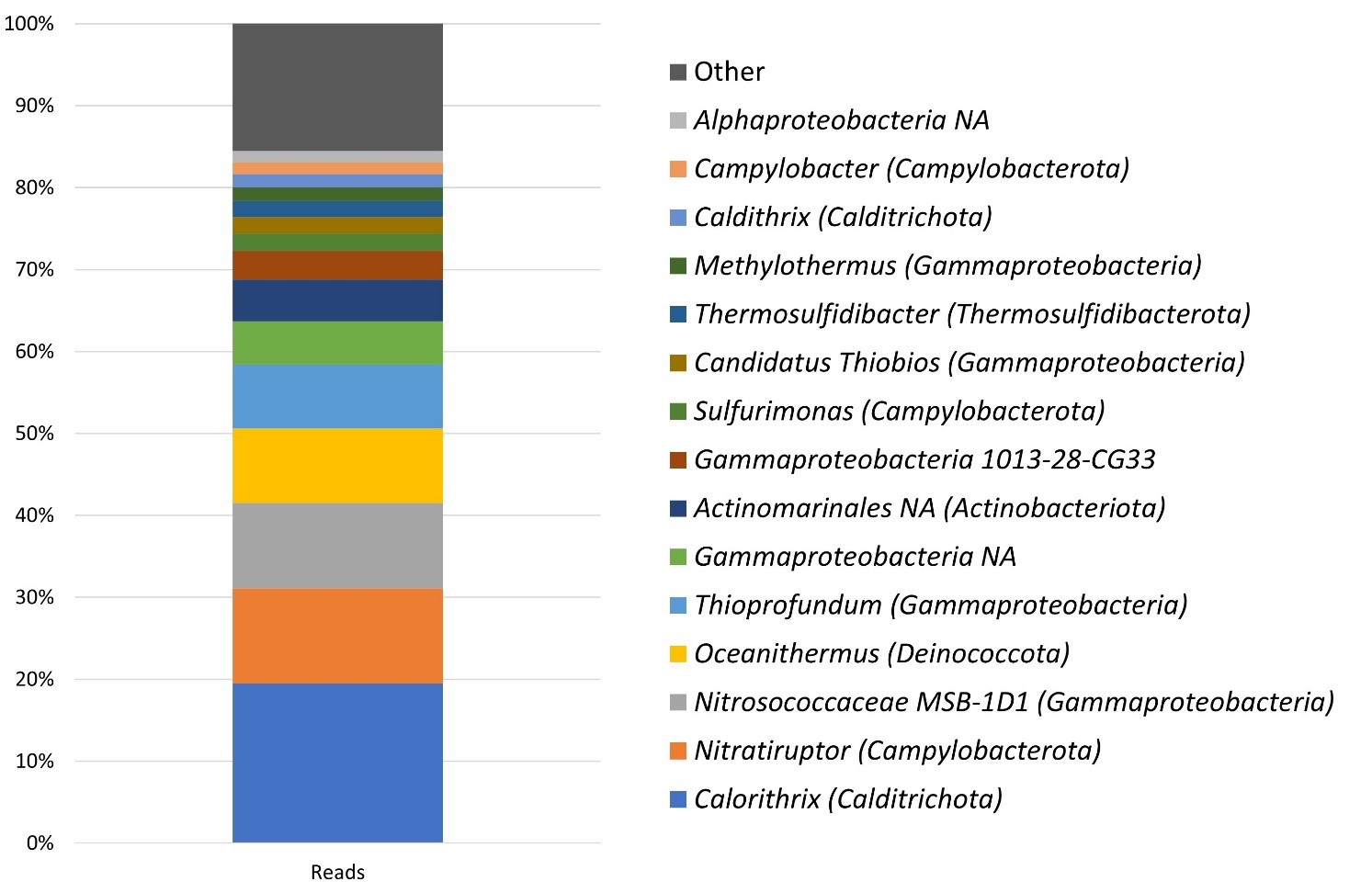


**Supplementary Figure 3.** Detection frequency of prokaryotic genera in the LC2 environmental sample. The corresponding phyla are also given in brackets.


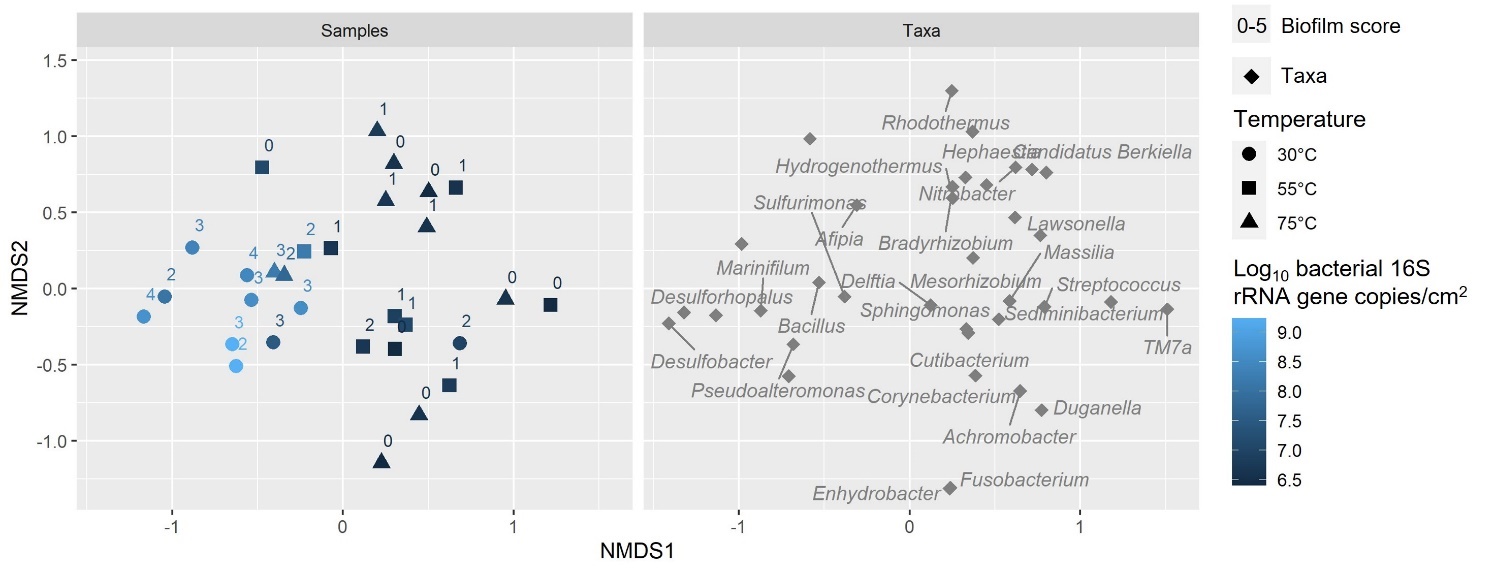


**Supplementary Figure 4.** Figure 6B untruncated. Non-Metric Multidimensional Scaling (NMDS) biplot based on the relative abundances of genera and Bray-Curtis dissimilarities. Taxa data points without labels correspond to ASVs of unassigned genera.

# Current consumption


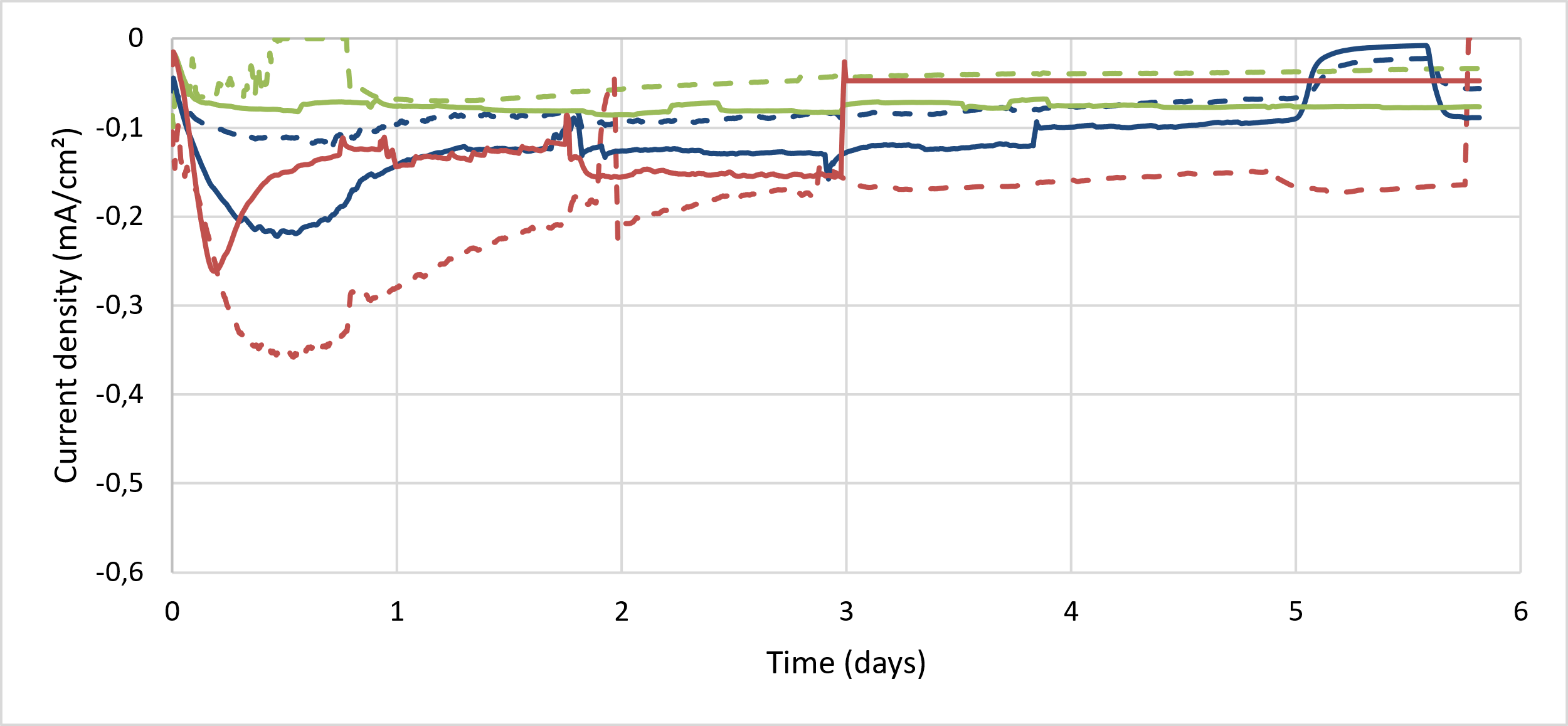

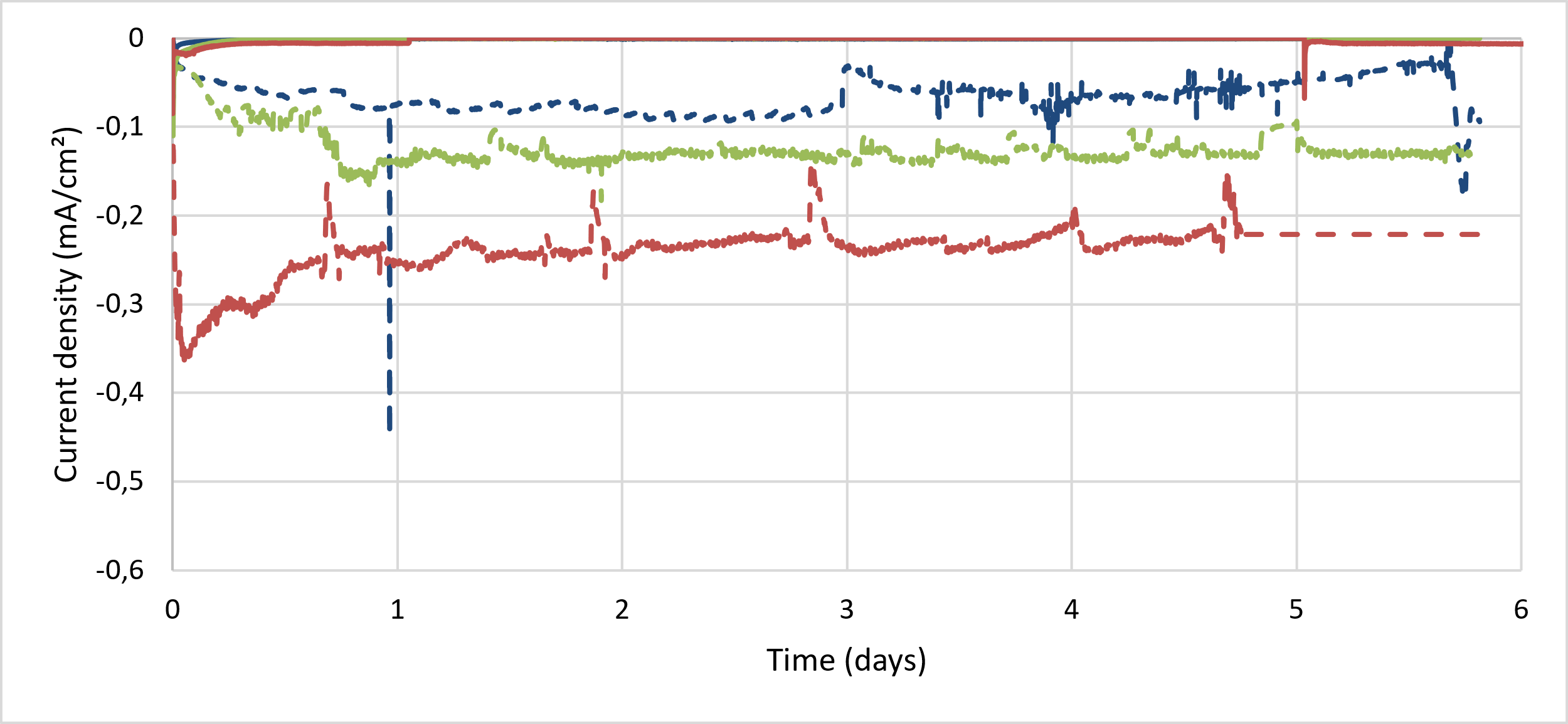

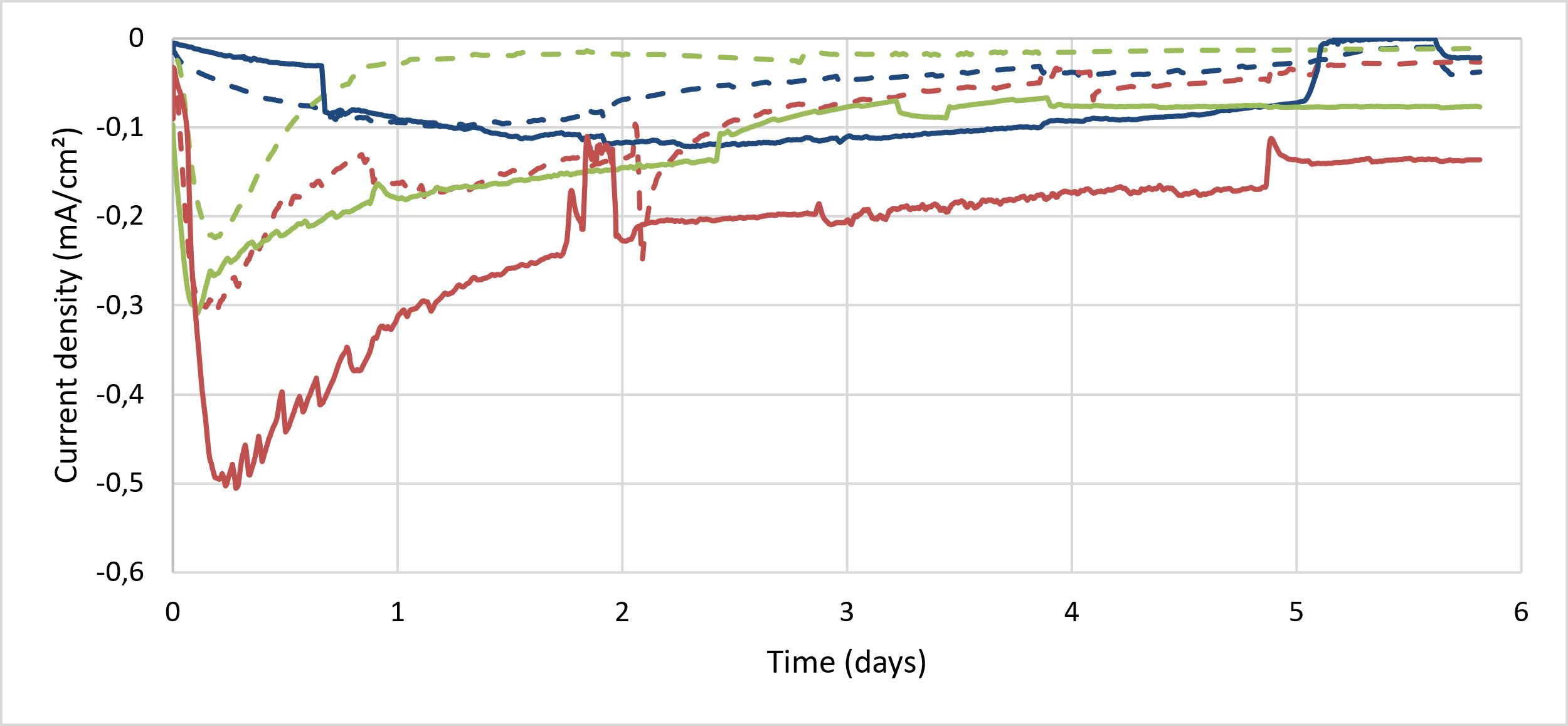

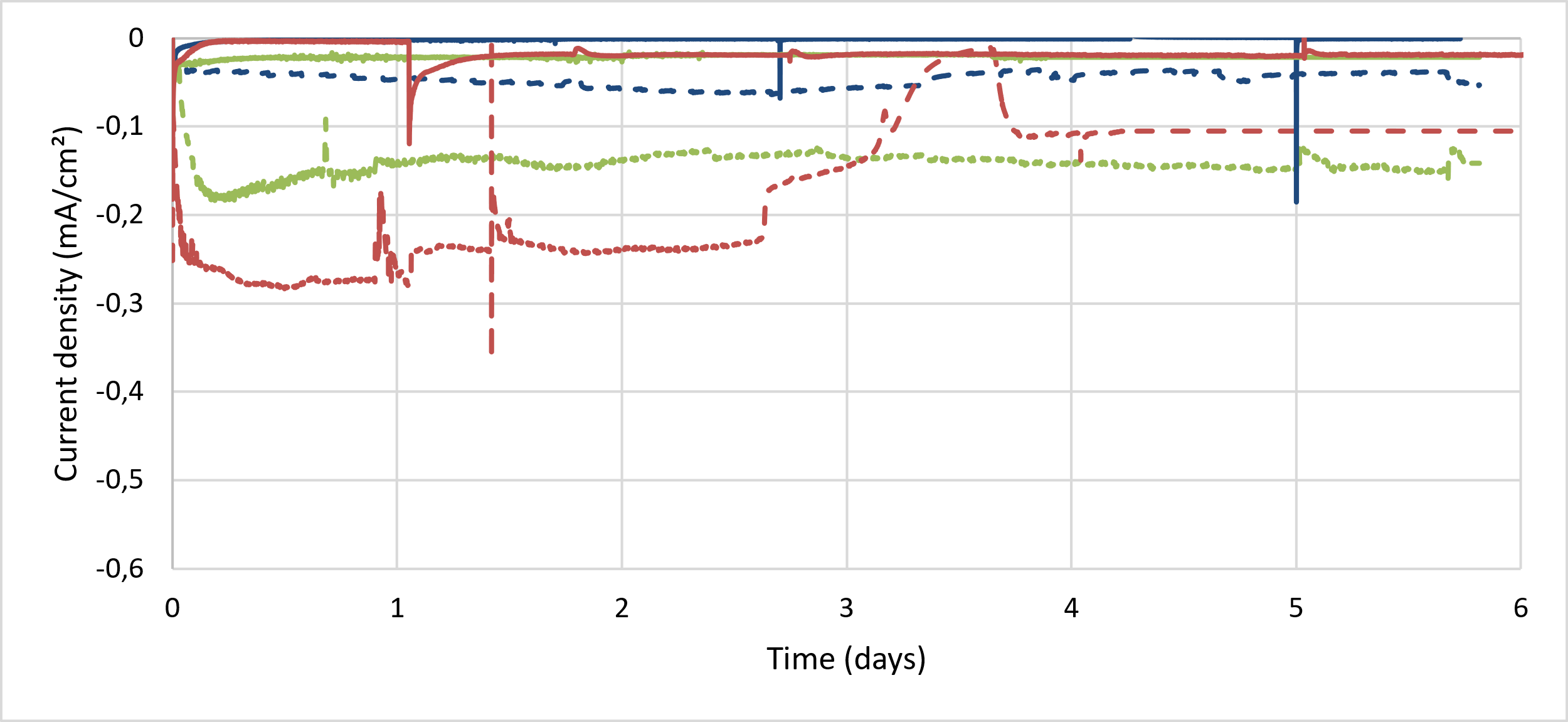

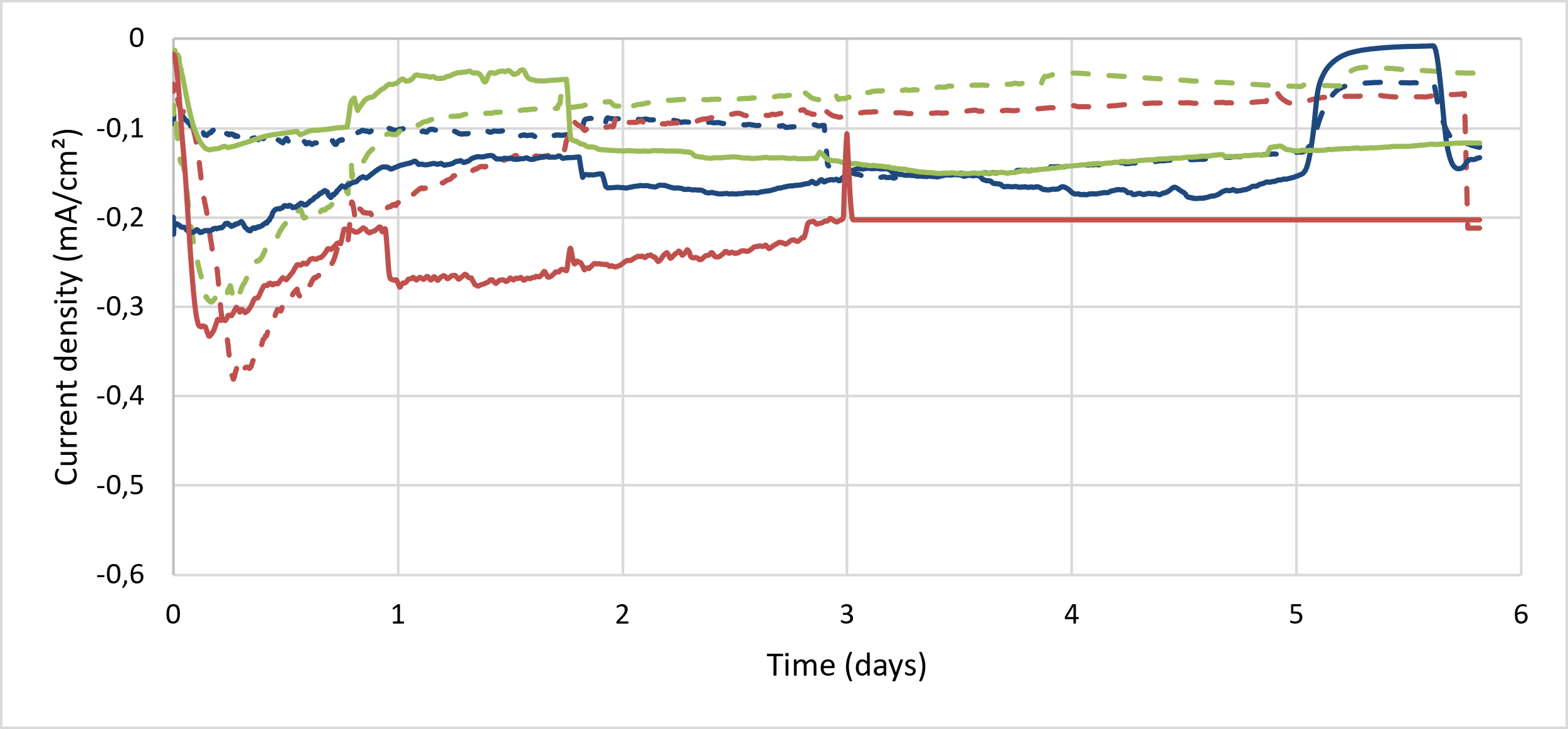

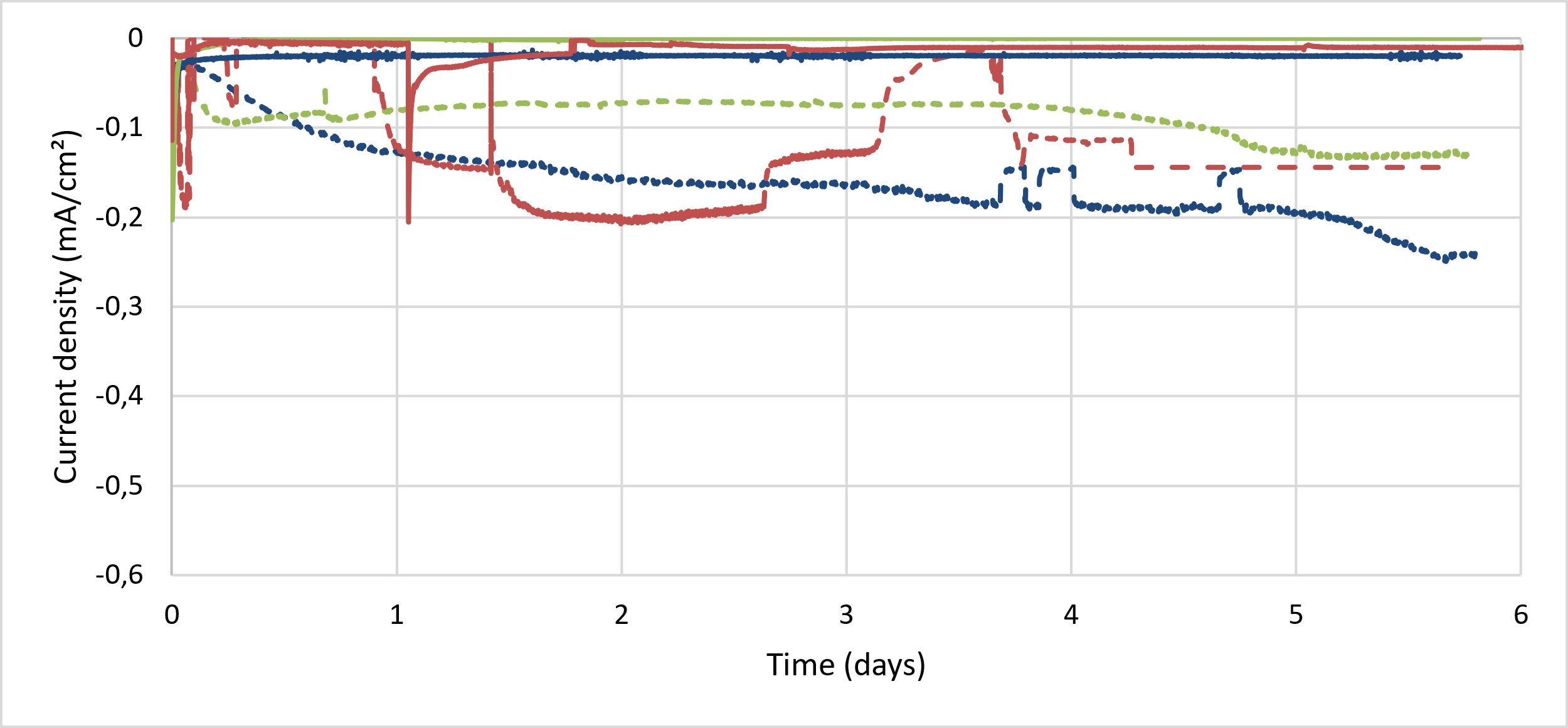

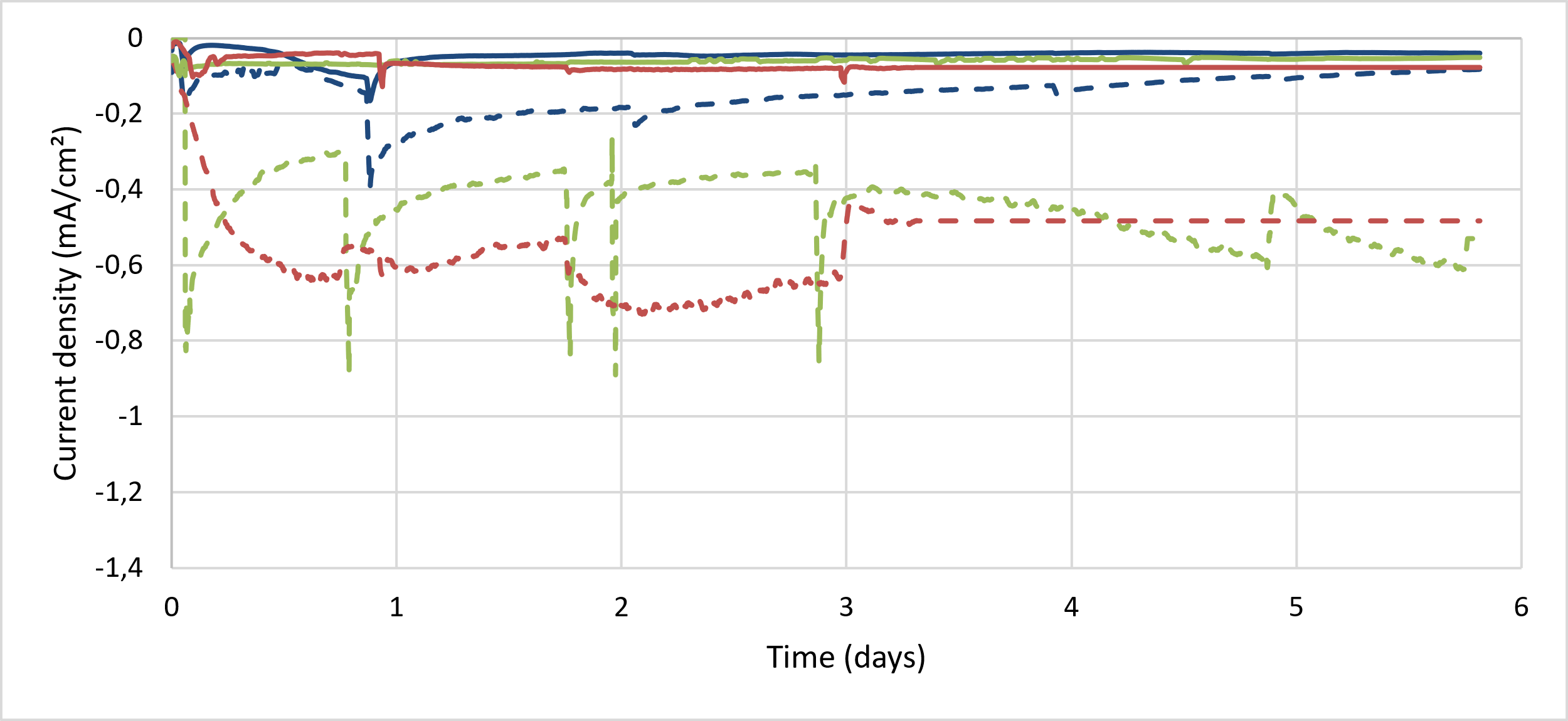

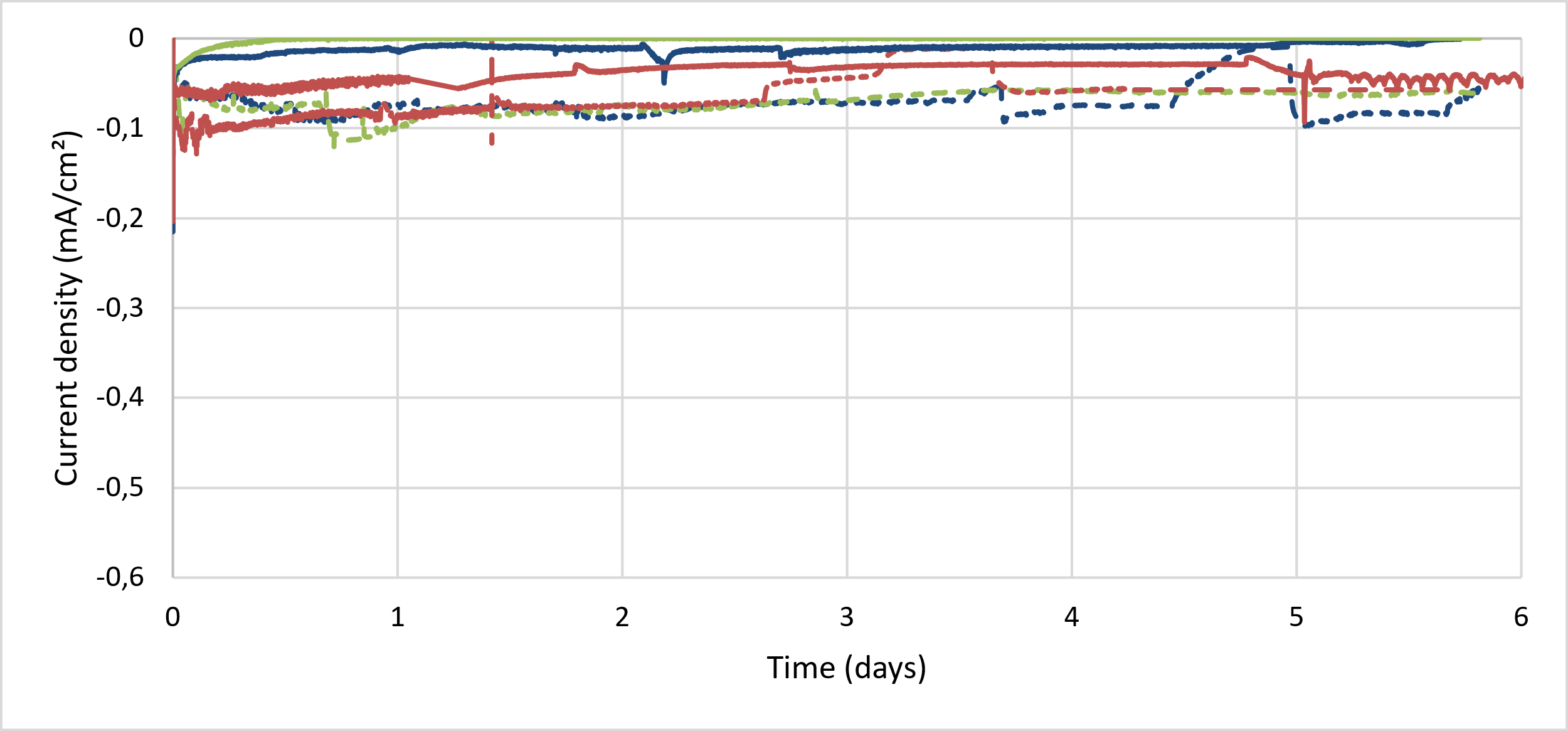

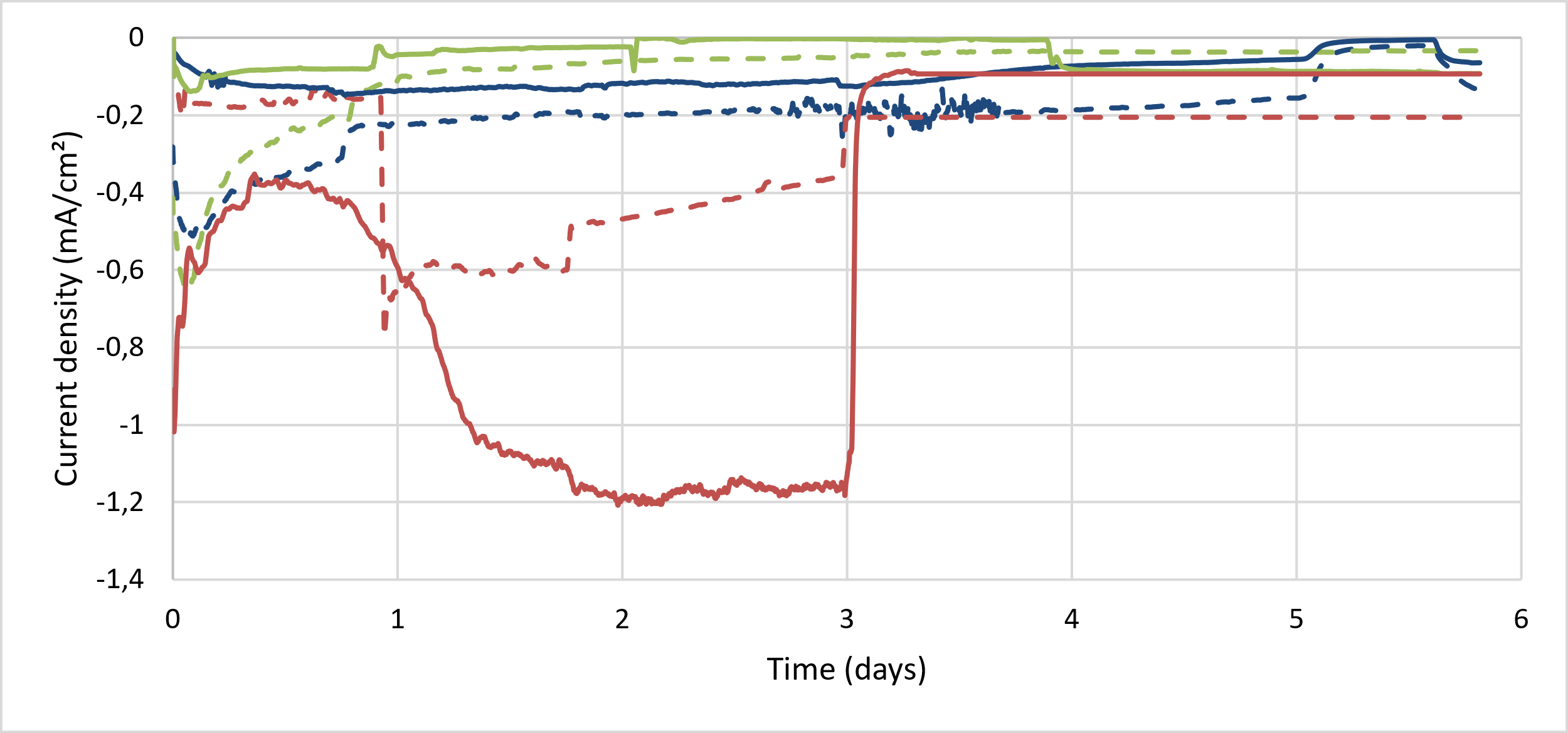

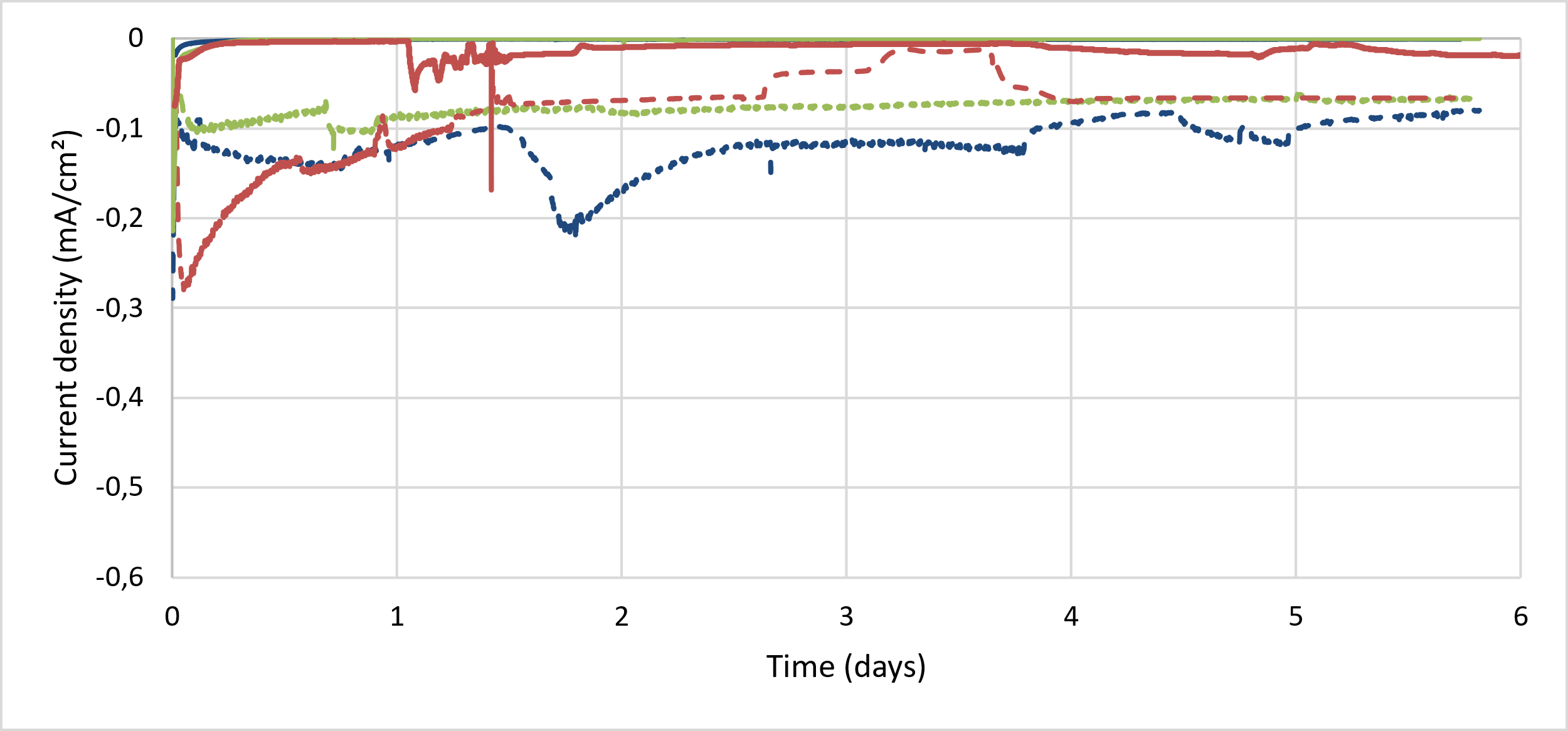


BJ – SO_4_

BJ – O_2_

BJ – NO_3_

BJ – FeOx

BJ – CO_2_

LC2 – SO_4_

LC2 – O_2_

LC2 – NO_3_

LC2 – FeOx

LC2 – CO_2_

**Supplementary Figure 5.** Current evolution over the experiments during the chronoamperometry polarized at -600 mV vs SHE.

# COD evolution


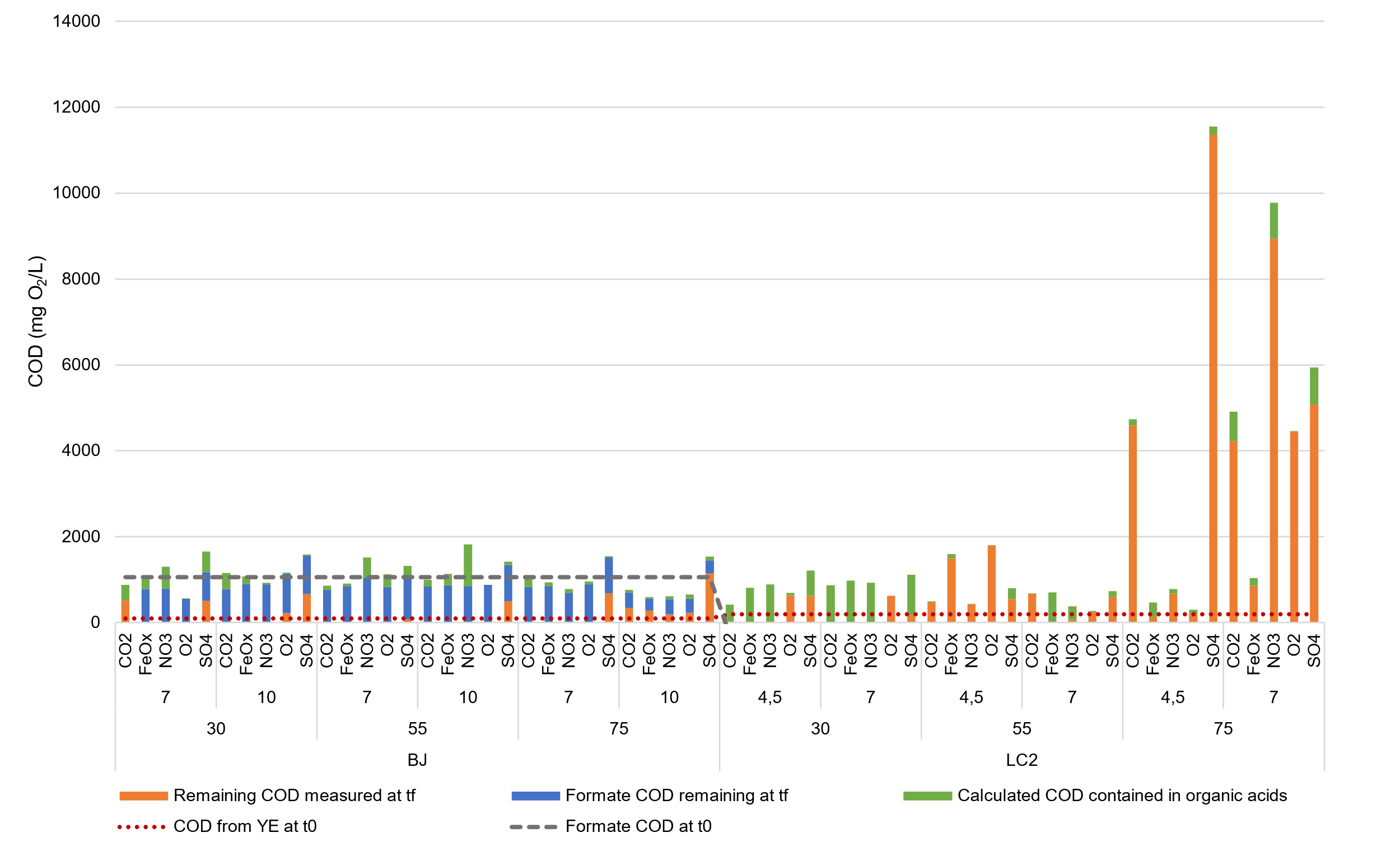


**Supplementary Figure 6.** Chemical Oxygen Demand of the medium calculated at the initial time (t0) provided by the yeast extract (red dotted line) and the formate (grey dashed line) and measured at the final time (tf) and divided into remaining formate, organic acid produced and the COD from the remaining biomass and/or yeast extract.

# Hydrogen Evolution Reaction

**Supplementary Table 1.** Standard potential (V vs SHE) of H_2_ evolution reaction for the minimum H_2_ concentration threshold for metabolic activity of different metabolisms at different pH and temperature. Calculated based on coefficient from Steven G. Bratsch; Standard Electrode Potentials and Temperature Coefficients in Water at 298.15 K. J. Phys. Chem. Ref. Data 1 January 1989; 18 (1): 1–21. <https://doi.org/10.1063/1.555839>.

|  |  |  | Temperature | | |
| --- | --- | --- | --- | --- | --- |
|  |  | H_2_ threshold concentrations* | 30°C | 55°C | 75°C |
| pH 4.5 | Aerobic respiration | 0.35 nM | -0.079 | -0.087 | -0.094 |
|  | Denitrification | 0.015 nM | -0.038 | -0.043 | -0.047 |
|  | Iron Reduction | 0.03 nM | -0.047 | -0.053 | -0.057 |
|  | Sulfate Reduction | 0.7 nM | -0.088 | -0.097 | -0.104 |
|  | Methanogenesis | 0.4 nM | -0.081 | -0.089 | -0.096 |
| pH 7 | Aerobic respiration | 0.35 nM | -0.229 | -0.250 | -0.266 |
|  | Denitrification | 0.015 nM | -0.188 | -0.206 | -0.22 |
|  | Iron Reduction | 0.03 nM | -0.197 | -0.216 | -0.23 |
|  | Sulfate Reduction | 0.7 nM | -0.239 | -0.26 | -0.277 |
|  | Methanogenesis | 0.4 nM | -0.231 | -0.252 | -0.269 |
| pH 10 | Aerobic respiration | 0.35 nM | -0.410 | -0.445 | -0.474 |
|  | Denitrification | 0.015 nM | -0.369 | -0.401 | -0.427 |
|  | Iron Reduction | 0.03 nM | -0.378 | -0.411 | -0.437 |
|  | Sulfate Reduction | 0.7 nM | -0.419 | -0.455 | -0.484 |
|  | Methanogenesis | 0.4 nM | -0.412 | -0.447 | -0.476 |
|  |  | |  |  |  |

* According to Karadagli, F., Marcus, A., & Rittmann, B. E. (2023). Microbiological hydrogen (H_2_) thresholds in anaerobic continuous-flow systems: Effects of system characteristics. *Biotechnology and Bioengineering*, 120, 1844–1856. <https://doi.org/10.1002/bit.28415>.
